# Sensing In Exergames for Efficacy and Motion Quality: Scoping Review of Recent Publications

**DOI:** 10.2196/52153

**Published:** 2024-11-05

**Authors:** Sebastian Dill, Philipp Niklas Müller, Polona Caserman, Stefan Göbel, Christoph Hoog Antink, Thomas Tregel

**Affiliations:** 1 KIS*MED (AI Systems in Medicine) Technical University of Darmstadt Darmstadt Germany; 2 Serious Games Research Group Technical University of Darmstadt Darmstadt Germany

**Keywords:** exergame efficacy, motion quality assessment, vital signs, body sensors, camera, virtual reality

## Abstract

**Background:**

Many studies have shown a direct relationship between physical activity and health. It has also been shown that the average fitness level in Western societies is lower than recommended by the World Health Organization. One tool that can be used to increase physical activity for individual people is exergaming, that is, serious games that motivate players to do physical exercises.

**Objective:**

This scoping review of recent studies regarding exergame efficacy aims to evaluate which sensing modalities are used to assess exergame efficacy as well as motion quality. We also analyze how the collected motion sensing data is being leveraged with respect to exergame efficacy and motion quality assessment.

**Methods:**

We conducted 2 extensive and systematic searches of the ACM Digital Library and the PubMed database, as well as a single search of the IEEE Xplore database, all according to the PRISMA (Preferred Reporting Items for Systematic Reviews and Meta-Analyses) statement. Overall, 343 studies were assessed for eligibility by the following criteria: The study should be peer-reviewed; the year of publication should be between 2015 and 2023; the study should be available in English or German; the study evaluates the efficacy of at least 1 exergame; sensor data is recorded during the study and is used for evaluation; and the study is sufficiently described to extract information on the exergames, sensors, metrics, and results.

**Results:**

We found 67 eligible studies, which we analyzed with regard to sensor usage for both efficacy evaluation and motion analysis. Overall, heart rate (HR) was the most commonly used vital sign to evaluate efficacy (n=52), while the Microsoft Kinect was the most commonly used exergame sensor (n=26). The results of the analysis show that the sensors used in the exergames and the sensors used in the evaluation are, in most cases, mutually exclusive, with motion quality rarely being considered as a metric.

**Conclusions:**

The lack of motion quality assessment is identified as a problem both for the studies and the exergames themselves since incorrectly executed motions can reduce an exergame’s effectiveness and increase the risk of injury. Here we propose how to use the same sensors both as input for the exergame and to assess motion quality by presenting recent developments in motion recognition and sensing.

## Introduction

Exergames are interactive games with the additional goal of engaging players in physical activity to promote a healthy lifestyle and increase players’ physical fitness [1,2]. Increasing availability of commercial sensing technologies has allowed exergames such as Beat Saber (Beat Games) [3] for various virtual reality (VR) systems and Ring Fit Adventure (Nintendo) for Nintendo Switch [4] to reach a large audience and become highly commercially successful, selling more than 4 million and 14 million copies, respectively [5,6]. Typically, these exergames use sensing technologies as input devices to either control an in-game avatar or detect the execution of specified motions. However, previous studies on motion-based games suggest that exergames should also give feedback on the motion’s quality and provide specific guidelines to follow [7,8]. Without these measures, players may not perform the demanded motions correctly, leading to diminished health benefits and risk of injury [9].

A common research question with regards to exergames is that of their efficacy. Efficacy refers to the effectiveness or ability of exergames to achieve their intended goals or their desired effect. This goal can be defined as an increase in the participants’ performance, physical activity, or motivation [10]. Previous reviews and meta-analyses on exergames generally confirm the existence of positive effects associated with exergames [10-13]. Nevertheless, the reviews primarily assert that exergames are most effective in facilitating light- to moderate-intensity physical activity, and only a small proportion of exergames have demonstrated the ability to significantly increase physical activity levels among users [14-16]. Furthermore, previous reviewers have primarily focused on assessing the efficacy of exergames in specific populations, such as children and adolescents without [11,12,16] or explicitly with adults with overweight [13] or older adults [15,17-19]. Only a few reviews encompass a broader range of participants and do not specifically focus on any particular population [14].

The evaluation process, particularly sensor technology usage, is typically not the focus of existing reviews. Hence, this paper investigates recent studies on the efficacy of exergames to identify which sensing technologies are used both in the exergames themselves and in their evaluation. We further review whether current exergames and their evaluations include any motion quality assessment. Motion Quality as a term is not clearly defined and is often visually evaluated by a professional physiotherapist or sports scientist. One of the most comprehensive approaches to defining the term is given by Skjaerven et al [20], who found that motion quality has many different aspects, including biomechanical as well as physiological and temporal characteristics. Therefore, motion quality assessment requires evaluating the motion of all relevant body parts at every point in time. Since we want to focus on the study design and methodology, with little regard for results or target group, we opt for a scoping review approach. For each study, we assess how the sensing data is being leveraged with respect to exergame efficacy and motion quality assessment. Based on our findings, we discuss how state-of-the-art methods for assessing motion quality and already used sensing technologies could be used to improve the efficacy of exergames and reduce the risk of injury during play.

## Methods

### Overview

The goal of this scoping review was to identify sensing modalities in recent studies that evaluate exergame efficacy. For this, 3 systematic searches were conducted in accordance with the PRISMA (Preferred Reporting Items for Systematic Reviews and Meta-Analyses) statement [21] and PRISMA-ScR (Preferred Reporting Items for Systematic Reviews and Meta-Analyses extension for Scoping Reviews) [22]. A PRISMA-ScR checklist is provided in Multimedia Appendix 1. The database searches were conducted at different points in time by one of the researchers, each without a detailed review protocol.

### Search Strategy and Search Terms

Due to the interdisciplinary nature of the research field, 3 different databases were included in the review. To cover both computer science and clinical research, the ACM Digital Library and the subject-specific PubMed database were searched on September 14, 2022, and again on July 14, 2023. A final search on the engineering-focused IEEE Xplore database was conducted on January 15, 2024.

We defined 4 main requirements for studies to be included in our review. First, studies have to feature the evaluation of an exergame. Second, the evaluation has to be conducted quantitatively with sensors with regard to the game’s efficacy. Third, the games should focus on general fitness or sports to avoid studies focusing on activities of daily living. Finally, the studies should be recent, which we defined as being published between 2015 and 2023. Based on these requirements, relevant search terms were identified, combined, and generalized as follows:

(exergam* OR “fitness game”)

AND (efficacy OR evaluat* OR “heart rate” OR vo2 OR oxygen)

AND (fitness OR sport)

For ACM Digital Library, the search terms were searched for in the categories “Title,” “Abstract,” and “Author Keyword,” each category connected with an OR operator, whereas for PubMed and IEEE Xplore, the search terms were typed “as is” into the “Query Box” and “Command Search,” respectively. A detailed search strategy is given in Multimedia Appendix 2.

### Study Eligibility Criteria and Selection

Afterward, the following eligibility criteria based on the previously stated requirements were defined. The results were screened and filtered accordingly:

1. Publication type: Peer-reviewed study
2. Publication year: 2015 to 2023
3. Available in English or German
4. The study evaluates the efficacy of at least one exergame
5. Sensor data is recorded during the study and used for evaluation
6. The study is sufficiently described to extract information on the exergames, sensors, metrics, and results

The studies were split among the authors to be screened by abstract and assessed for eligibility. The results of this screening process were then presented and discussed conjointly.

### Data Extraction and Data Analysis

Eligible studies were evaluated with regard to their general study design (including participant numbers and focus group), their evaluation methods (including evaluation metrics, vital signs, and sensor usage), and the exergames used (including exergame sensors as well as additional motion sensing). The results were again discussed, assessed for relevance for the review, and generalized conjointly.

## Results

### Overview

The first search yielded 253 studies of which 30 were removed before the screening process as they were duplicates or not available in English or German. An additional 31 studies were identified through previous work of included authors and citation searching, resulting in a total of 258 studies included in the screening process. 208 studies were excluded in this screening process based on the eligibility criteria stated above, leaving a total of 49 eligible studies. The second search yielded an additional 40 studies, of which 32 were excluded from the screening process. The final search, conducted on the IEEE Xplore database, yielded another 50 studies. After screening by title, abstract, and eligibility criteria, 10 studies were deemed fitting, resulting in a total of 67 studies that were included in our review.

**Figure 1:**
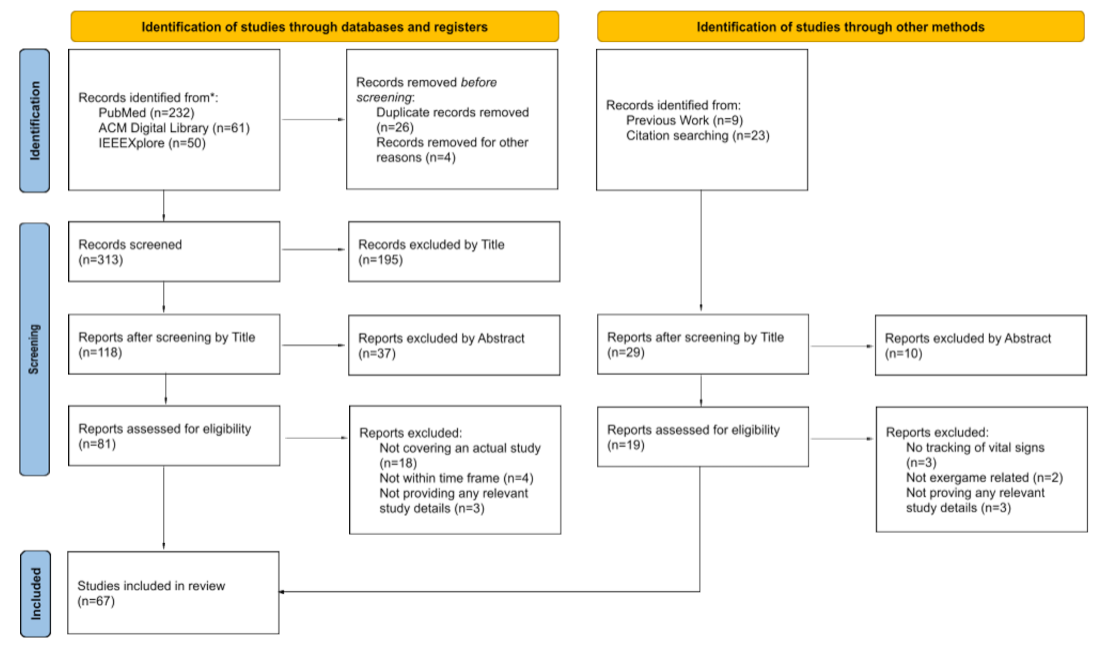
PRISMA (Preferred Reporting Items for Systematic Reviews and Meta-Analyses) [21] flow diagram of the systematic review process.

Table 1 gives a brief summary of the vital sign-sensing statistics. Multimedia Appendix 3 [23-89] and Multimedia Appendix 4 [23,24,26-58,60-90] feature additional tables that present details for all 67 studies included in the review. Multimedia Appendix 3 provides an overview of the study design, participants, and how efficacy was assessed. Participation numbers ranged from 6 [23] to 360 [24], with a median of 28. There was a large variety of different focus groups, with the biggest group being healthy adults (n=19 studies). Multimedia Appendix 4 details the exergames and corresponding sensors used in each study and gives information on additional motion sensing if there were any. Not all studies explicitly mention all the exergames evaluated; some feature the evaluation of a multitude of different games, and some tested games they developed themselves. Overall, the 67 studies included in our review feature the evaluation of approximately 49 different exergames. The overall review process with all screening steps is outlined in the PRISMA flow diagram in Figure 1.

**Table 1:** Summary of vital sign sensing statistics. Detailed statistics for each study can be found in Multimedia Appendix 3.

	Measured vital signs				Evaluated metrics							Evaluation criteria analyzed over time			
	HR^a^	VO_2_	Other	mean HR		peak HR or % max HR	peak VO_2_ or % max VO_2_	MET^b^	EE^c^	Other	Performance		Intensity or PA^d^	Motivation	Other
Number of studies	52	22	18	35		31	21	16	15	45	18		11	6	10
Proportion of studies, %	78	33	27	52		46	31	24	22	67	27		16	9	15

^a^HR: heart rate.

^b^MET: metabolic equivalent of task.

^c^EE: energy expenditure.

^d^PA: physical activity.

### Vital Sign Sensing Technologies

As per our inclusion criterion, all 67 studies aim to assess efficacy. For that, 54 studies used sensors to measure vital signs, while 11 studies relied only on motion sensing, and 2 studies assessed efficacy without any sensors but with standardized tests only. The majority, that is, 52 of the 54, measured the player’s heart rate (HR), and 22 measured the oxygen uptake (VO_2_) (more details in Table 1). Furthermore, 18 studies measured other vital signs such as blood pressure, blood lactate concentration, and carbon dioxide production.

### Efficacy Assessment

In the context of exergames, efficacy refers to the effectiveness or ability of exergames to achieve their intended goals or their desired effect under optimal or controlled conditions. Efficacy is distinguished from effectiveness, with efficacy focusing on how well an approach works in ideal conditions, while effectiveness considers its performance in real-world or practical situations [91]. However, this distinction is not always clearly made by the researchers [25]. Therefore, in the context of this review, the 2 terms are used interchangeably. Typical goals for exergames can be defined as an increase in the participants’ performance, physical activity, or motivation [10,92]. Exergame efficacy is usually evaluated by analyzing these metrics over time or in comparison with conventional physical exercises. Often, they are estimated by measuring vital signs, doing standardized tests, or filling out questionnaires. Efficacy is a highly individual metric and will vary for different activities, different target demographics, and from person to person [10].

When looking at the studies’ approaches to assessing exergame efficacy, several different study designs were identified. First of all, a distinction can be made by whether the studies evaluate a single group or compare 2 or more groups. The former is referred to as a within-subject or cross-over study. The latter category is referred to as controlled studies, which again can be split into 2 subcategories. Randomized controlled studies compare randomly split groups, while cross-section studies compare nonrandomized groups based on preexisting conditions. The studies can also be categorized by how many measurements are done. Single-measurement studies use measured metrics and questionnaires to compare the participants’ performance, motivation, and general physical activity with nongaming-related exercises. Alternatively, studies with the repeated-measures design, focusing on the long-term effects of exergaming, do the same measurements more than once to observe longitudinal developments. The latter approach was used by 22 studies, while the other 45 studies focused on the first approach. Details on the study design of every included study can be found in Multimedia Appendix 3.

### Acute Effects

A total of 53 out of the 67 (79%) studies focus on efficacy evaluation by measuring vital signs in their studies. Out of all analyzed studies (more details in Table 1), 35 reported mean HR, and 31 reported peak HR or proportionate maximum HR (%max HR), with the maximum HR commonly being age-predicted. Ventilation-based evaluation took place in 21 analyzed studies, reporting peak VO_2_ or proportionate maximum VO_2_ (%max VO_2_).

Based on the recorded vital signs, 15 studies processed their participants’ data to indicate the recorded energy expenditure (EE) for the exergame activity. In total, 16 studies calculated and reported the metabolic equivalent of task (MET) values for each activity. A total of 8 studies overlapped with studies reporting EE. Furthermore, noteworthy vital-sign-based evaluation metrics are systolic and diastolic blood pressure [26-29], heart rate reserve [30-33], pulse wave velocity with total peripheral resistance and stroke volume [34], respiratory exchange rate [35,36], lactate concentration [37,38], maximum power output [39], muscle activity [40], concentration through EEG (electroencephalography) [41]. In 3 studies, other derived indicators are used: play intensity and hemodynamic reactivity are calculated based on players’ heart rate [37], and the so-called activity counts are based on accelerometer measurements [42,43]. Many studies used subjective questionnaire scales for assessment, which we do not evaluate as they are unrelated to vital sign sensing.

### Long-Term Effects

Although many analyzed studies (35 out of 67) comprised more than 1 session of playing exergames, only a minority evaluated changes over time (22 out of 67), and thus qualifies as a longitudinal study in the context of this paper. The evaluation time for longitudinal studies ranged from 4 weeks [44-47] to 6 months [48].

As detailed in Multimedia Appendix 3, most researchers analyzed players’ performance. The performance evaluation was predominantly done objectively by tracking changes in anaerobic [46,49,50] or muscular fitness [51,52], functional strength [50], peak VO_2_ [28,48], postural control [45], reaction time [24], or cognitive function [53]. The performance was sometimes evaluated subjectively using exercise self-efficacy score [54].

Other evaluation metrics analyzed over time include changes in intensity, usually based on HR or EE [29,45,55,56], whereas further research publications evaluated playtime for different physical activity levels [28,48,51]. In addition to performance and intensity, 6 research publications analyzed changes in motivation, such as play frequency and duration [56] as well as flow [45], player engagement [55] or enjoyment [54], and happiness [57].

### Motion Sensing Technologies

The 67 reviewed studies assess various exergames that use different motion sensing technologies, among which the Kinect for Xbox 360 (Microsoft Corp) [93] is the most commonly used (26 out of 67). The Kinect combines a regular RGB (red-green-blue) camera with a depth sensor and can thus be considered a camera-based system. In addition, 1 study each included an exergame using the EyeToy (London Studio) for PlayStation 2 [54] and PS Move (Sony Interactive Entertainment) for PlayStation 3 [44], resulting in 28 out of 67 studies featuring at least one camera-based system. Ergometers, dance mats, balance boards, and other sensing technologies specialized for specific motions are used in 21 out of 67 studies and, thus, make up the second-most common type of motion sensing technology.

VR systems and body-worn inertial measurement units (IMUs) are featured less commonly. Only 5 out of 67 studies feature body-worn IMUs for motion sensing, and only one of them uses IMUs not included in a smartphone or game controller. Furthermore, 7 studies evaluate exergames that use the Nintendo Wii remote controllers held in the player’s hands. These controllers are not body-worn and combine an IMU and an infrared sensor with an external emitter for tracking. Together with VR systems, which were used in 16 out of 67 studies as either standalone or within the so-called ExerCube (Sphery AG) [94], they make up the category of hybrid sensing (overall n=22). Finally, one study [58] features a game that does not use any form of motion sensing.

Out of the 67 studies, 21 did additional motion sensing during the exergaming for evaluation purposes. Common motion sensors used for this task are body-worn accelerometers and IMUs (used in 12 studies) and force plates (used in 4 studies). Furthermore, 2 studies used hybrid motion-capture systems, and 2 used camera-based systems. A specialized system to measure reaction times, an exercise bike, surface electromyography, and a handgrip dynamometer were used in 1 study each.

### Motion Quality Assessment

Motion quality, as a term, is not clearly defined and is often visually evaluated by a professional physiotherapist or sports scientist. One of the most comprehensive approaches to defining the term is given by Skjaerven et al [20], where they investigated the lived experiences of a group of expert physiotherapists in search of essential characteristics and features of the term. They found that motion quality has many different aspects, including the biomechanical “characteristics of path and form in [motion]” as well as the physiological and temporal “characteristic of flow, elasticity, vitality, and rhythm in [motion].” Therefore, to provide specific feedback on the full motion quality, it would be necessary to assess the whole motion by tracking and evaluating the motion of all relevant body parts at every point in time, independent from the gameplay.

An essential part of exergaming is the game’s ability to track and analyze the player’s motions to judge the player’s gameplay and their exercise quality. Often, exergames focus on the gameplay aspect, only evaluating motions implicitly by requiring players to interact with the virtual environment, for example, slashing, collecting, or avoiding virtual objects [3,39,40,95,96]. Alternatively, some games combine both evaluations by having players fit a predefined shape [59,97,98]. While such an approach might be suitable to verify if players are performing a specific motion at all, it only enables detecting static poses or certain joints without analyzing the holistic motion execution.

None of the 49 games featured in the studies explicitly assess the quality of exercises performed. However, 7 studies use additional motion sensing to do some form of quality assessment during their evaluation. In addition, 4 of these 7 studies focus on specific motion aspects, such as assessing the angular displacement or range of motion of certain joints [41,55], quantifying postural sway [45], and evaluating shoulder flexibility [26]. Only 3 studies [44,51,60] assess the players’ motion quality as a whole, using a multitude of sensors and either a standardized test or a professional physiotherapist. However, for 2 of the studies, these assessments do not happen during the exergame but are used to identify general motion quality at times when the subjects are not playing. The final study [44] employs an expert to do an analysis of gross upper body biomechanics based on video recordings of the subjects during the exergames. Therefore, this is the only study in our review that fulfills the condition of holistically evaluating all relevant body parts at every point in time during the exercise, which we consider necessary for motion quality assessment, according to Skjaerven et al [20]. However, this is not an automated process and instead requires manual input from an expert.

## Discussion

### Overview

To the best of our knowledge, this review represents the first work analyzing sensor usage in studies that examine exergame efficacy. We found 67 eligible studies, which we analyzed with regard to sensor usage for both efficacy evaluation and motion analysis. Overall, HR was the most commonly used vital sign to evaluate efficacy (n=52), while the Microsoft Kinect was the most commonly used exergame sensor (n=26). The results of the analysis show that the sensors used in the exergames and the sensors used in the evaluation are, in most cases, mutually exclusive.

### Principal Findings

From the review, 2 main conclusions can be drawn: First, the majority of papers focus on measuring vital signs to infer a physical activity metric. Second, an overall lack of motion quality assessment can be stated among exergame studies. For the studies, this lack of quality assessment can lead to undetected and unwanted influences on the results. For example, it might be hard to differentiate if a lack of efficacy should be attributed to the exergame or to the participants’ execution, especially in longitudinal studies. Furthermore, it also poses a problem for the exergames themselves since the players are incentivized to optimize for score, which might lead to optimal gameplay that does not coincide with a correct exercise execution and might lead to injuries instead of increased health [9]. Therefore, in the following outlook, we outline how different types of sensor technologies could be used with existing motion analysis techniques to assess motion quality in exergames.

### Outlook

#### Quality Assessment Based on Cameras

##### Overview

While the traditional approach of motion analysis by a camera is a hybrid one with visual markers attached to certain body parts, a lot of recent research has focused on markerless methods solely based on single-camera video images [99]. This gives cameras a few advantages over other sensing methods: They are unobtrusive to the players and allow them a full range of motion; they can be easily set up and moved around, and they are comparably cheap and widely available [99]. The recent approaches can be differentiated by their modality: While classic RGB cameras are mostly used when no depth information is needed, so-called RGB-D (red-green-blue-depth) cameras like the Microsoft Kinect [93] are able to also record depth information [100,101].

##### RGB-D

As stated, 26 of the 28 exergames considered in the review that used cameras for the gaming input relied on the Kinect RGB-D camera. In addition to the advantages of cameras already listed, the Kinect also has its origin in gaming, making it a widespread tool not only for scientists but also for game developers. However, all papers in our review use the Kinect only as input for the games, not for an explicit motion quality assessment. This is surprising as, outside of the exergaming context, RGB-D cameras are used in several motion research areas, such as gait analysis [102-104] and fall detection [105], where they have shown to be a reliable tool to capture and evaluate full-body movements. Sporadically, the Kinect has been used in exergaming-related motion analysis as well, albeit not for quality control in an efficacy assessment study. Examples include motion dissimilarity analysis [106] or interrater reliability evaluation between a Kinect system [107] and a human rater.

##### RGB

In contrast, classic RGB cameras are not commonly used as motion sensors in exergaming, aside from rare examples [108]. This is also apparent from our review since only 2 exergames used an RGB camera compared to the 26 using the Kinect. However, studies without exergame context have shown that modern RGB-based systems are well-suited to do quantitative motion analysis. Systems like MonoCap (Zhejiang University) [109], OpenPose (Carnegie Mellon University) [110], and MediaPipe Pose (Google) [111] have proven that full-body pose estimation can be done with high accuracy. One of the most well-researched applications of RGB-based motion analysis is gait analysis, which can either be done with a feature-based approach using pose estimation [112-114] or a feature-less approach directly on the images [115,116]. More complex medical applications such as joint load prediction [117] and motion limitation analysis [118] indicate the method’s ability to do precise quality assessment of human movements. Furthermore, RGB cameras have already been used in sports analysis [119]. These use cases imply the suitability of RGB cameras as a tool for both gaming input and quality assessment in exergaming.

#### Quality Assessment Based on Hybrid Sensing Techniques

The release of consumer-grade virtual reality systems contributed to the development of many immersive exergames. An analysis of the 29 top VR exergames from a recent review [120] shows that players prefer games providing a high level of exertion (equivalent to real-world exercise level), whereas a high level of immersion is important for distraction (reducing perceived exertion). Most reviewed exergames using hybrid sensing technology indeed provide a playful fitness experience; nevertheless, existing approaches often fail to analyze motion execution to detect errors or to provide specific feedback on motion quality. For example, approaches letting players fit a predefined shape [59,97,98] might be suitable to verify if players are performing a specific motion at all. However, they only enable the detection of static poses without analyzing motion execution. Furthermore, many VR exergames enabling interaction with VR objects usually depend only on hand-held devices and often lack lower body movement.

To overcome this limitation, other researchers use additional off-the-shelf VR sensors. Previous studies already concluded that VR sensors are feasible for clinical, research, and industry usage [121,122]. For example, Martin-Niedecken et al [90,123] demonstrated how trackers attached to wrists and ankles can be used to recognize different exercises, such as burpees, lunges, and punches. The motion quality, presented by a star ranking, is then assessed according to how well players reached predetermined target points and how quickly they returned to their initial pose. A further study [124] also analyzes the entire motion execution of different yoga poses and thereby identifies execution errors.

Another possibility to assess motion quality is marker-based motion capture systems, for example, OptiTrack (NaturalPoint Inc.) [125] and Vicon Tracker (Vicon Motion Systems Ltd.) [126]. These systems are the gold standard for tracking individual joint positions and angular movements with high accuracy and low latency [127,128]. However, as such systems require several markers and cameras, they can be obtrusive and not suitable for home or clinical environments. Nevertheless, previous research publications have already demonstrated that motion capture suits are reliable for analyzing holistic motion executions during dance [129], identifying exercise execution errors during physical exercise [130], or conducting kinematic trunk motion analysis [60].

#### Quality Assessment Based on Body-Worn Sensors

IMUs consisting of an accelerometer, a gyroscope, and sometimes a magnetometer are the most common types of body-worn sensors. They are integrated into commercial devices such as smartphones, smartwatches, and fitness trackers. Among other things, they are often used to track fitness activities and activities of daily living. Compared with camera-based or hybrid approaches, they are particularly well suited to track nonstationary activities. In the following, we will not discuss other body-worn sensors, such as EMG sensors, because of their limited availability and applicability.

While regular body-worn IMUs do not provide as extensive motion data as camera-based or hybrid systems, exergames could use them as robust, low-cost sensors to assess specific quality metrics and enable tracking without a stationary setup. An example of this can be seen in the commercially successful exergame Ring Fit Adventure (Nintendo) for the Nintendo Wii, which tracks and assesses performed exercises through IMUs placed at the thigh and the inside of an elastic ring held by the user. Alternatively, commercial IMU-based systems such as Xsens (Movella) [131], Noraxon Ultium Motion (Noraxon) [132], or the Teslasuit (Teslasuit, Deep Divers Ltd) [133] could be used for an extensive motion analysis in a mobile setting [130].

Whereas some exergames in our reviewed papers use controllers that incorporate an IMU, none rely purely on body-worn sensors for tracking. In 7 papers [41,43,51,60-64], body-worn IMUs are used to assess physical activity. Only Ko et al [41] and Mueller et al [60] additionally use body-worn IMU data for quality assessment by determining the users’ range of motion and back posture respectively.

In their review, Rana and Mittal [134] show that wearable sensors can be successfully deployed for kinematic analysis in a variety of sports applications. While these included sports applications such as swimming [135,136], which are ill-suited for exergames, most sports applications presented could be integrated into exergames. Particularly noteworthy applications are swing sports such as tennis [137-143] and badminton [140,144], in which IMUs can either be wrist-worn or integrated into the racket to track and assess individual swings, as it can be difficult to track these with stationary setups in a practical setting.

### Limitations

This scoping review is prone to the same search-related limitations as other reviews of this type. First, only articles published in international journals and full articles published in conference proceedings written in English or German are considered. Therefore, potentially relevant studies published in other languages may have been missed. Second, some articles were excluded from the analysis because the required information to assess their eligibility based on the inclusion criteria was not provided. Third, we assess a lack of reproducibility for the ACM Digital Library. In general, a very high volatility can be noted in the amount of records ACM’s search function returns when changing individual words or operators. Since the current search results in less records than the original search, which were all included in the review, we consider this to be a minor limitation. Finally, out of the 67 studies included in this scoping review, 8 studies were not found firsthand using the search terms but instead through previous work of included authors and citation searching. This may indicate that there would have been even more fitting search terms for the review question. However, due to the author’s experience with the topic and the unambiguity of the results, we are confident that we were able to mitigate any possible negative effects and that the included studies present a complete overview of the current state of research.

In addition to limitations related to the search, we can also note 2 limitations related to the scope of evaluation. They are a deliberately chosen result of our research focus, which means to analyze the studies’ methodology with regards to sensing instead of their results. First, the definitions for the terms “efficacy” and “motion quality” used in this paper focus on how these 2 metrics are evaluated and do not go into detail on what qualifies as “good” efficacy or motion quality. Instead, we evaluate how well the studies are able to assess efficacy and motion quality with the methodology and especially the sensors they use. Therefore, we do not define what a “high/low quality motion” may look like as this question is highly specific to the individual motion and therefore cannot be answered generally. For the same reason, we also do not go into detail on the cohorts’ age, sex, and focus group as they are predominantly relevant to the studies’ results.

### Conclusions

In this paper, we conduct a scoping review of recent studies that examine exergame efficacy to determine which sensors are being used and how they are being used during gameplay and evaluation. Our results show that most studies evaluate exergame efficacy by measuring vital signs, the most common being heart rate (52 out of 67) and oxygen consumption (22 out of 67). However, motion quality is only assessed in 7 out of 67 studies despite being an important factor in an exergame’s effectiveness and risk of injury. Furthermore, out of the 49 exergames evaluated in the reviewed studies, none feature quality assessment during or after gameplay, and only 3 studies feature motion quality assessment beyond the exergame’s feedback.

Since exergames already use motion sensing technologies to track the player’s motions, they could also be used for external quality assessment. Therefore, we discuss recent advances in the field of motion analysis and potential use cases of different sensors commonly used in exergames. We come to the conclusion that many of the same sensing technologies typically used in exergames and exergame studies are well-suited for additional motion quality assessment to ensure consistent exergame effectiveness and reduce the likelihood of injury while exergaming.

## Appendix

Multimedia Appendix 1 Preferred reporting items for systematic reviews and meta-analyses extension for scoping reviews (PRISMA-ScR) checklist.

Multimedia Appendix 2 Search strategies and results for individual databases.

Multimedia Appendix 3 Summary of studies with regards to the Evaluation of Exergame Efficacy.

Multimedia Appendix 4 Summary of studies with regards to the Evaluation of Games and Motion Sensing.

## Abbreviations

EE: energy expenditure
EEG: electroencephalography
HR: heart rate
IMU: inertial measurement unit
MET: metabolic equivalent of task
PRISMA: Preferred Reporting Items for Systematic Reviews and Meta-Analyses
PRISMA-ScR: Preferred Reporting Items for Systematic Reviews and Meta-Analyses extension for Scoping Reviews
RGB: red-green-blue
RGB-D: red-green-blue-depth
VO2: oxygen uptake
VR: virtual reality
