# Supplementary material for: Sensing In Exergames for Efficacy and Motion Quality: Scoping Review of Recent Publications
Source: JMIR Serious Games. 2024 Nov 5;12:e52153. doi: 10.2196/52153 (PMC11576609; doi:10.2196/52153)
Supplement: Multimedia Appendix 2 [file games_v12i1e52153_app2.docx]

Table S1: Search Strategies and Results for Individual Databases.

| Database | Date Searched | Search Strategy | Results |
| --- | --- | --- | --- |
| [ACM Digital Library](https://dl.acm.org/search/advanced) | 14.09.2022 | "query": { Title:((exergam* OR "fitness game") AND (efficacy OR evaluat* OR "heart rate" OR vo2 OR oxygen) AND (fitness OR sport)) OR Abstract:((exergam* OR "fitness game") AND (efficacy OR evaluat* OR "heart rate" OR vo2 OR oxygen) AND (fitness OR sport)) OR Keyword:((exergam* OR "fitness game") AND (efficacy OR evaluat* OR "heart rate" OR vo2 OR oxygen) AND (fitness OR sport)) }  "filter": { E-Publication Date: (01/01/2015 TO *) } | 50 records |
| [ACM Digital Library](https://dl.acm.org/search/advanced) | 14.07.2023 | "query": { Title:((exergam* OR "fitness game") AND (efficacy OR evaluat* OR "heart rate" OR vo2 OR oxygen) AND (fitness OR sport)) OR Abstract:((exergam* OR "fitness game") AND (efficacy OR evaluat* OR "heart rate" OR vo2 OR oxygen) AND (fitness OR sport)) OR Keyword:((exergam* OR "fitness game") AND (efficacy OR evaluat* OR "heart rate" OR vo2 OR oxygen) AND (fitness OR sport)) }  "filter": { E-Publication Date: (01/01/2015 TO *) } | 61 records (including the previous 50 records) |
| [PubMed](https://pubmed.ncbi.nlm.nih.gov/advanced/) | 14.09.2022 | (exergam* OR "fitness game") AND (efficacy OR evaluat* OR "heart rate" OR vo2 OR oxygen) AND (fitness OR sport)  Filters applied: from 2015/1/1 - 2022 | 203 records |
| [PubMed](https://pubmed.ncbi.nlm.nih.gov/advanced/) | 14.07.2023 | (exergam* OR "fitness game") AND (efficacy OR evaluat* OR "heart rate" OR vo2 OR oxygen) AND (fitness OR sport)  Filters applied: from 2015/1/1 - 2023 | 232 records (including the previous 203 records) |
| [IEEEXplore](https://ieeexplore.ieee.org/search/advanced/command) | 15.01.2024 | ((exergam* OR "fitness game") AND (efficacy OR evaluat* OR "heart rate" OR vo2 OR oxygen) AND (fitness OR sport))  Filters Applied: 2015 - 2023 | 50 records |
