# Supplementary material for: Sensing In Exergames for Efficacy and Motion Quality: Scoping Review of Recent Publications
Source: JMIR Serious Games. 2024 Nov 5;12:e52153. doi: 10.2196/52153 (PMC11576609; doi:10.2196/52153)
Supplement: Multimedia Appendix 3 [file games_v12i1e52153_app3.docx]

Multimedia Appendix 3. Summary of Studies with regards to the Evaluation of Exergame Efficacy.

| Author | Sample Size | Focus Group | Study Design | Vital Signs | | | Evaluation Metric | | | | | | Evaluation criteria  analyzed over time | | | |
| --- | --- | --- | --- | --- | --- | --- | --- | --- | --- | --- | --- | --- | --- | --- | --- | --- |
|  |  |  |  | HR | VO2 | Other | mean HR | peak HR /  % max HR | peak VO2/  % max VO2 | MET | EE | Other | Performance | Intensity/ PA | Motivation | Other |
| Bronner et al. [56] | 14  (7m, 7f, 26.6±9.5 yo) | physically active adults | repeated-  measures | **+** | **+** | **+** | **+** |  |  | **+** | **+** |  |  | **+** | **+** |  |
| Wu et al. [119] | 17  (7m, 10f, 22.0±2.9 yo) | healthy adults | cross-over study | **+** | **+** |  | **+** | **+** | **+** | **+** | **+** |  |  |  |  |  |
| McGuire and Willems [36] | 10 (10m, 23±5 yo) | healthy adults | cross-over study | **+** | **+** |  |  |  | **+** | **+** | **+** | **+** |  |  |  |  |
| Hoffmann et al. [120] | 16  (9m, 7f, 29.6±8.2 yo) | physically active adults | non-repeated single-group | **+** |  |  | **+** | **+** |  |  |  |  |  |  |  |  |
| Rodrigues et al. [121] | 19  (19m, 20±2 yo) | healthy adults | cross-over study | **+** | **+** |  |  | **+** | **+** | **+** |  |  |  |  |  |  |
| Neves et al. [28] | 18  (9m, 9f, 23±5 yo) | healthy adults | cross-sectional study | **+** |  | **+** | **+** |  |  |  |  | **+** |  |  |  |  |
| Dos Santos et al. [55] | 40  (18m, 22f, 6-12 yo) | overweight children | cross-section repeated-  measures | **+** |  |  |  |  |  |  |  | **+** | **+** |  |  |  |
| Smits-  Engels-  man et al. [51] | 36  (18m, 18f, 6-10 yo) | children with develop-  mental coordination disorder | cross-section pre-post experimental design |  |  |  |  |  |  |  |  | **+** | **+** |  |  |  |
| Barry et al. [46] | 47  (27m, 20f, 33±12 yo) | physically active adults | randomized controlled two-arm trial design (not double-blind) | **+** |  |  | **+** | **+** |  |  |  | **+** | **+** | **+** | **+** | **+** |
| Mackintosh et al. [104] | 36  (19m, 17f, 21.7±3.8 yo) | adults | randomized cross-over | **+** |  |  |  |  |  |  | **+** |  |  |  |  |  |
| Benzing et al. [122] | 65  (65m, 14.5±1.1 yo) | healthy ado-lescents | randomized between-subjects design | **+** |  |  | **+** |  |  |  |  | **+** |  |  |  |  |
| Chung et al. [39] | 10  (5m, 5f, 24.4±6.8/ 20.6±3.2 yo) | elite rowing athletes | cross-over study | **+** | **+** | **+** | **+** |  | **+** |  |  | **+** |  |  |  |  |
| Moholdt et al. [123] | 8 (23.9±0.6 yo) | healthy adults | within-subject study | **+** |  | **+** | **+** | **+** |  |  |  | **+** |  |  |  |  |
| Patten et al. [61] | 20  (9m, 11f, 7±1.8 yo, 4-11 y) | children | cross-over study | **+** |  |  | **+** |  |  |  |  |  |  |  |  |  |
| Tietjen and Devereux [124] | 25  (13m, 12f, 22.9±6.4 yo) | healthy adults | Cross-over + repeated measures | **+** | **+** |  | **+** | **+** | **+** | **+** |  |  | **+** | **+** |  |  |
| Kaos et al. [57] | 72  (45m, 27f, 10 yo) | physically inactive children | controlled repeated measures study | **+** |  |  | **+** |  |  |  |  | **+** |  | **+** | **+** |  |
| Pasco et al. [44] | 163  (100m, 63f, 20.3±1.3 yo) | healthy adults | randomized controlled study |  |  |  |  |  |  | **+** |  | **+** |  |  |  |  |
| Farrow et al. [40] | 16 (8m, 8f, 22±4 yo) | healthy adults | cross-over study | **+** | **+** |  |  | **+** | **+** |  | **+** | **+** |  |  |  |  |
| McDough et al. [105] | 60  (30m, 30f, 23.6±4.1 yo) | healthy adults | cross-over study |  |  |  |  |  |  | **+** | **+** |  |  |  |  |  |
| Rodrigues et al. [125] | 19  (19m, 20.6±2 yo) | healthy adults | cross-over study | **+** | **+** |  | **+** | **+** | **+** | **+** |  |  |  |  |  |  |
| Polechoński et al. [32] | 30 (30m, 23.8±1.3 yo) | non- specific | cross-over study | **+** |  |  |  | **+** |  |  |  | **+** |  |  |  |  |
| Viana et al. [126] | 34 (34m, 26±4.9 yo) | non- specific | non-repeated single group |  | **+** | **+** |  |  | **+** |  | **+** |  |  |  |  |  |
| Feodoroff et al. [41] | 33 (33m, 23.9±4.6 yo) | adults | cross-over study | **+** |  | **+** |  |  |  |  |  | **+** |  |  |  |  |
| McDough et al. [106] | 20  (2m, 18f, 27.3±4.3 yo) | retired elite athletes | cross-over repeated measures study | **+** |  |  |  |  |  |  | **+** | **+** |  |  |  |  |
| Çakir-Atabek et al. [33] | 22 (22m, 20.6±2.2 yo) | adult athletes | cross-over repeated measures study | **+** | **+** | **+** | **+** |  | **+** | **+** | **+** | **+** |  |  |  |  |
| Roure et al. [127] | 60  (31m, 29f, 20.8±1.3 yo) | non- specific | randomized controlled study | **+** | **+** | **+** |  | **+** | **+** |  |  | **+** |  |  |  |  |
| Berg and Moholdt [128] | 10  (5m, 5f,  32±8/ 27±3 yo) | non- specific | non-repeated single group study | **+** | **+** | **+** |  | **+** | **+** |  |  |  |  |  |  |  |
| Berg et al. [49] | 52  (27m, 25f) | physically inactive adults | randomized controlled repeated measures study | **+** | **+** | **+** | **+** | **+** | **+** | **+** |  | **+** | **+** | **+** |  | **+** |
| Ko et al. [42] | 10 (28.5±6.1 yo) | healthy adults | cross-over study |  |  | **+** |  |  |  |  |  | **+** |  |  |  |  |
| Martin-Niedecken et al. [129] | 21 (12m, 9f, 5.3± 1.5 yo) | healthy adults | Randomized | **+** |  |  | **+** | **+** |  |  |  | **+** |  |  |  |  |
| Aygün and Çakir-Atabek [130] | 43  (22m, 21f, 21.7±1.9/ 20.8±2.5 yo) | non- specific | cross-over study | **+** | **+** | **+** | **+** | **+** | **+** | **+** | **+** | **+** |  |  |  |  |
| Ketelhut et al. [38] | 27  (14m, 13f, 25±4 yo) | healthy adults | Cross-sectional study | **+** | **+** | **+** | **+** | **+** | **+** | **+** | **+** | **+** |  |  |  |  |
| Comeras-Chueca et al. [52] | 29  (16m, 13f, 9-12 yo) | over-  weight and obese children | Randomized Controlled Trial |  |  |  |  |  |  | **+** |  | **+** | **+** | **+** |  | **+** |
| Berg et al. [29] | 30  (>= 18 yo) | physically inactive healthy adults | Randomized Controlled Trial | **+** | **+** | **+** |  | **+** | **+** | **+** |  | **+** | **+** | **+** | **+** | **+** |
| Badau et al. [23] | 360  (180m, 180f, 13.6±1.1 yo) | children athletes | Cross-sectional study |  |  |  |  |  |  |  |  | **+** | **+** |  |  |  |
| Soria Campo et al. [131] | 15  (10m, 5f, 23±7) | healthy children and adults | Randomized Cross-over Trial | **+** | **+** |  | **+** | **+** | **+** |  |  | **+** |  |  |  |  |
| Gouveia et al. [54] | 31  (9m, 22f, 61-78 yo) | elderly | Randomized Controlled Trial |  |  |  |  |  |  |  |  | **+** | **+** |  |  |  |
| Ketelhut et al. [132] | 28  (15m, 13f, 24.8±3.8 yo) | healthy adults | cross-over study | **+** | **+** | **+** | **+** | **+** | **+** |  |  | **+** |  |  |  |  |
| Li et al. [27] | 23  (7m, 16f, 72.5±6) | elderly | Randomized Controlled Trial | **+** |  |  |  | **+** |  |  |  | **+** |  |  |  |  |
| Kircher et al. [35] | 28  (15m, 13f, 24.8±3.9 yo) | healthy adults | cross-over study | **+** |  |  | **+** | **+** |  |  |  | **+** |  |  |  |  |
| Martin-Niedecken et al. [92] | 40  (21m, 19f, 34.4±8.7 yo) | non- specific | within-subjects | **+** |  |  | **+** | **+** |  |  |  |  |  |  |  |  |
| Marks et al. [133] | 15  (8m, 7f, 21.3±1.4 yo) | healthy adults | Repeated measures design | **+** | **+** |  | **+** | **+** | **+** |  | **+** |  |  |  |  |  |
| Evans et al. [34] | 36  (18m, 18f, 18-30 yo) | non- specific | Repeated measures design | **+** |  |  |  |  |  |  |  | **+** |  |  |  |  |
| Sousa et al. [43] | 29  (20m, 9f, 23.2±2.1 yo) | young adults with low levels of physical activity | Repeated measures design | **+** |  |  | **+** | **+** |  |  |  | **+** |  |  |  |  |
| Wu et al. [47] | 80  (46m, 34f, 23.2±3 yo) | young adults | Randomized | **+** |  |  | **+** |  |  |  |  | **+** | **+** |  |  | **+** |
| Monedero et al. [134] | 23  (11m, 12f, 24.8±1 yo) | healthy young adults | Cross-over | **+** | **+** |  | **+** | **+** | **+** | **+** | **+** |  |  |  |  |  |
| Dębska et al. [135] | 61  (51m, 10f) | healthy adults | Randomized or cross-section + cross-over | **+** |  |  | **+** | **+** |  |  |  |  |  |  |  |  |
| Ketelhut et al. [50] | 34  (17m, 17f, 10.5±0.7 yo) | children | Randomized | **+** |  |  | **+** | **+** |  |  |  | **+** | **+** |  |  | **+** |
| Lisón et al. [31] | 62  (28m, 34f, 11.8±1.3 yo) | healthy children | Cross-over | **+** |  |  | **+** |  |  |  |  | **+** |  |  |  |  |
| Stewart et al. [136] | 32  (16m, 16f, 22.6±2.6 yo) | young adults (<=39 yo) | Cross-over | **+** |  |  | **+** |  |  |  |  | **+** |  | **+** | **+** |  |
| Mueller et al. [67] | 13  (5m, 8f, 41±16 yo) | patients with chronic lower back pain | Repeated measures |  |  |  |  |  |  |  |  | **+** |  |  |  |  |
| Ciążyńska et al. [37] | 93,  (45m, 48f, 21.69±2.76 yo) | healthy young adults | Cross-over | **+** |  | **+** | **+** | **+** |  |  | **+** | **+** | **+** |  |  |  |
| Lin et al. [53] | 35  (10m, 25f, 64.1±6.8 yo) | older adults | Randomized | **+** |  |  |  | **+** |  |  |  | **+** | **+** |  |  | **+** |
| Lin et al. [107] | 17  (9m, 8f, 18-50 yo) | healthy adults | Randomized |  |  |  |  |  |  |  |  | **+** |  |  |  |  |
| Wounda et al. [137] | 24  (22m, 2f, 42±13 yo) | wheel- chair-  bound patients | Randomized | **+** | **+** |  |  | **+** | **+** |  |  | **+** |  |  |  |  |
| Mologne et al. [30] | 32  (18m, 14f, 24.3 yo) | healthy young adults | Cross-sectional | **+** | **+** | **+** |  |  | **+** |  |  | **+** | **+** | **+** |  | **+** |
| Hastürk and Munusturlar [58] | 22  (22m, 2f, 31.7 ± 3.94 yo) | young adults | Repeated measures |  |  |  |  |  |  |  |  | **+** | **+** |  |  | **+** |
| Sheu et al. [48] | 10  (62-81 yo) | older adults | Randomized Controlled Trial; between-  groups |  |  |  |  |  |  |  |  |  | **+** |  |  |  |
| Rosly et al. [45] | 13  (9m, 4f, 37±12 yo) | patients with spinal cord injury | between-groups | **+** |  |  | **+** | **+** |  |  |  |  |  | **+** | **+** |  |
| Wünsche et al. [22] | 6  (5m, 1f,  24±5 yo) | young adults | Single group study | **+** |  |  | **+** | **+** |  |  |  |  |  |  |  |  |
| Muñoz et al. [138] | 33  (10m, 23f, 67.1±4.2 yo) | older adults | within and between subjects | **+** |  |  |  |  |  |  | **+** | **+** |  |  |  |  |
| Cardona et al. [139] | 17 (3m, 14f, 64.5± 6.4 yo) | older adults | repeated measures design | **+** |  | **+** | **+** |  |  | **+** |  |  |  |  |  |  |
| Liu et al. [140] | 36  (22.31± 2.65 yo) | young adults (no athletes) | cross-over study | **+** |  |  | **+** |  |  |  |  |  |  |  |  |  |
| Liu et al. [140] | 42  (23.75±  2.64 yo) | young adults (no athletes) | randomized controlled study |  |  |  |  |  |  |  |  |  | **+** | **+** |  |  |
| Martin Dantas et al. [141] | 32 (8.30±0.67 for control, 8.73±0.76 for game) | Children without physical or functional limitation | Randomized |  |  |  |  |  |  |  |  | **+** | **+** |  |  | **+** |
| Han et al. [142] | 12  (8m, 4f, 20-45 yo) | Adults | randomized controlled study | **+** |  |  | **+** |  |  |  |  |  |  |  |  |  |
| Julainjatsono et al. [143] | 24  (15m, 9w, 20.08 yo) | non- specific | Non-repeated single-group study |  |  |  |  |  |  |  |  | **+** |  |  |  |  |
| Total |  |  |  | 52 | 22 | 18 | 35 | 31 | 21 | 16 | 15 | 45 | 18 | 11 | 6 | 10 |

Abbreviations: HR, Heart Rate; VO2, Oxygen Uptake; EE, Energy Expenditure; MET, Metabolic Equivalent of Task; PA, Physical Activity; yo, year old

References

[22] B. C. Wünsche et al., ‘Rift Racers - Effect of Balancing and Competition on Exertion, Enjoyment, and Motivation in an Immersive Exergame’, in 2021 36th International Conference on Image and Vision Computing New Zealand (IVCNZ), Dec. 2021, pp. 1–6. doi: 10.1109/IVCNZ54163.2021.9653159.

[23] D. Badau et al., ‘The Impact of Implementing an Exergame Program on the Level of Reaction Time Optimization in Handball, Volleyball, and Basketball Players’, Int. J. Environ. Res. Public. Health, vol. 19, no. 9, Art. no. 9, Jan. 2022, doi: 10.3390/ijerph19095598.

[25] A. G. Singal, P. D. R. Higgins, and A. K. Waljee, ‘A Primer on Effectiveness and Efficacy Trials’, Clin. Transl. Gastroenterol., vol. 5, no. 1, p. e45, Jan. 2014, doi: 10.1038/ctg.2013.13.

[27] J. Li, L. Li, P. Huo, C. Ma, L. Wang, and Y. L. Theng, ‘Wii or Kinect? A Pilot Study of the Exergame Effects on Older Adults’ Physical Fitness and Psychological Perception’, Int. J. Environ. Res. Public. Health, vol. 18, no. 24, Art. no. 24, Jan. 2021, doi: 10.3390/ijerph182412939.

[28] L. E. D. S. Neves et al., ‘Cardiovascular Effects of Zumba® Performed in a Virtual Environment Using Xbox Kinect’, J. Phys. Ther. Sci., vol. 27, no. 9, pp. 2863–2865, 2015, doi: 10.1589/jpts.27.2863.

[29] J. Berg, G. Haugen, A. I. Wang, and T. Moholdt, ‘High-Intensity Exergaming for Improved Cardiorespiratory Fitness: A Randomised, Controlled Trial’, Eur. J. Sport Sci., vol. 22, no. 6, pp. 867–876, Jun. 2022, doi: 10.1080/17461391.2021.1921852.

[30] M. S. Mologne et al., ‘The Efficacy of an Immersive Virtual Reality Exergame Incorporating an Adaptive Cable Resistance System on Fitness and Cardiometabolic Measures: A 12-Week Randomized Controlled Trial’, Int. J. Environ. Res. Public. Health, vol. 20, no. 1, Art. no. 1, Jan. 2023, doi: 10.3390/ijerph20010210.

[31] J. F. Lisón et al., ‘Competitive active video games: Physiological and psychological responses in children and adolescents’, Paediatr. Child Health, vol. 20, no. 7, pp. 373–376, Oct. 2015, doi: 10.1093/pch/20.7.373.

[32] J. Polechoński, M. Dębska, and P. G. Dębski, ‘Exergaming Can Be a Health-Related Aerobic Physical Activity’, BioMed Res. Int., vol. 2019, p. e1890527, Jun. 2019, doi: 10.1155/2019/1890527.

[33] H. Çakir-Atabek, C. Aygün, and B. Dokumacı, ‘Active Video Games Versus Traditional Exercises: Energy Expenditure and Blood Lactate Responses’, Res. Q. Exerc. Sport, vol. 91, no. 2, pp. 188–196, Apr. 2020, doi: 10.1080/02701367.2019.1653431.

[34] E. Evans, K. E. Naugle, A. S. Kaleth, B. Arnold, and K. M. Naugle, ‘Physical Activity Intensity, Perceived Exertion, and Enjoyment During Head-Mounted Display Virtual Reality Games’, Games Health J., vol. 10, no. 5, pp. 314–320, Oct. 2021, doi: 10.1089/g4h.2021.0036.

[35] E. Kircher et al., ‘Acute Effects of Heart Rate-Controlled Exergaming on Vascular Function in Young Adults’, Games Health J., vol. 11, no. 1, pp. 58–66, Feb. 2022, doi: 10.1089/g4h.2021.0196.

[36] S. McGuire and M. E. Willems, ‘Physiological Responses During Multiplay Exergaming in Young Adult Males are Game-Dependent’, J. Hum. Kinet., vol. 46, pp. 263–271, Jul. 2015, doi: 10.1515/hukin-2015-0054.

[37] J. Ciążyńska and J. Maciaszek, ‘Effects of Low-Immersive vs. High-Immersive Exercise Environment on Postural Stability and Reaction and Motor Time of Healthy Young Adults’, J. Clin. Med., vol. 12, no. 1, Art. no. 1, Jan. 2023, doi: 10.3390/jcm12010389.

[38] S. Ketelhut et al., ‘Gaming Instead of Training? Exergaming Induces High-Intensity Exercise Stimulus and Reduces Cardiovascular Reactivity to Cold Pressor Test’, Front. Cardiovasc. Med., vol. 9, 2022, doi: 10.3389/fcvm.2022.798149.

[39] L. M. Y. Chung, F. H. Sun, and C. T. M. Cheng, ‘Physiological and Perceived Responses in Different Levels of Exergames in Elite Athletes’, Games Health J., vol. 6, no. 1, pp. 57–60, Feb. 2017, doi: 10.1089/g4h.2016.0074.

[40] M. Farrow, C. Lutteroth, P. C. Rouse, and J. L. J. Bilzon, ‘Virtual-Reality Exergaming Improves Performance During High-Intensity Interval Training’, Eur. J. Sport Sci., vol. 19, no. 6, pp. 719–727, Jul. 2019, doi: 10.1080/17461391.2018.1542459.

[41] B. Feodoroff, I. Konstantinidis, and I. Froböse, ‘Effects of Full Body Exergaming in Virtual Reality on Cardiovascular and Muscular Parameters: Cross-Sectional Experiment’, JMIR Serious Games, vol. 7, no. 3, p. e12324, Aug. 2019, doi: 10.2196/12324.

[42] J. Ko, S.-W. Jang, H. T. Lee, H.-K. Yun, and Y. S. Kim, ‘Effects of Virtual Reality and Non–Virtual Reality Exercises on the Exercise Capacity and Concentration of Users in a Ski Exergame: Comparative Study’, JMIR Serious Games, vol. 8, no. 4, p. e16693, Oct. 2020, doi: 10.2196/16693.

[43] C. V. Sousa et al., ‘Active video games in fully immersive virtual reality elicit moderate-to-vigorous physical activity and improve cognitive performance in sedentary college students’, J. Sport Health Sci., vol. 11, no. 2, pp. 164–171, Mar. 2022, doi: 10.1016/j.jshs.2021.05.002.

[44] D. Pasco, C. Roure, G. Kermarrec, Z. Pope, and Z. Gao, ‘The Effects of a Bike Active Video Game on Players’ Physical Activity and Motivation’, J. Sport Health Sci., vol. 6, no. 1, pp. 25–32, Mar. 2017, doi: 10.1016/j.jshs.2016.11.007.

[45] M. M. Rosly, M. Halaki, H. M. Rosly, N. Hasnan, R. Husain, and G. M. Davis, ‘Arm Exercises for Individuals with Spinal Cord Injury: Exergaming versus Arm Cranking’, in 2019 IEEE 7th International Conference on Serious Games and Applications for Health (SeGAH), Aug. 2019, pp. 1–7. doi: 10.1109/SeGAH.2019.8882460.

[46] G. Barry, P. van Schaik, A. MacSween, J. Dixon, and D. Martin, ‘Exergaming (Xbox KinectTM) Versus Traditional Gym-Based Exercise for Postural Control, Flow and Technology Acceptance in Healthy Adults: A Randomised Controlled Trial’, BMC Sports Sci. Med. Rehabil., vol. 8, no. 1, p. 25, Aug. 2016, doi: 10.1186/s13102-016-0050-0.

[47] Y.-S. Wu et al., ‘Effect of the Nintendo Ring Fit Adventure Exergame on Running Completion Time and Psychological Factors Among University Students Engaging in Distance Learning During the COVID-19 Pandemic: Randomized Controlled Trial’, JMIR Serious Games, vol. 10, no. 1, p. e35040, Mar. 2022, doi: 10.2196/35040.

[48] F.-R. Sheu, Y.-L. Lee, H.-T. Hsu, and N.-S. Chen, ‘Effects of Gesture-Based Fitness Games on Functional Fitness of the Elders’, in 2015 IEEE 15th International Conference on Advanced Learning Technologies, Jul. 2015, pp. 158–160. doi: 10.1109/ICALT.2015.35.

[49] J. Berg, A. I. Wang, S. Lydersen, and T. Moholdt, ‘Can Gaming Get You Fit?’, Front. Physiol., vol. 11, 2020, doi: 10.3389/fphys.2020.01017.

[50] S. Ketelhut, L. Röglin, A. L. Martin-Niedecken, C. R. Nigg, and K. Ketelhut, ‘Integrating Regular Exergaming Sessions in the ExerCube into a School Setting Increases Physical Fitness in Elementary School Children: A Randomized Controlled Trial’, J. Clin. Med., vol. 11, no. 6, Art. no. 6, Jan. 2022, doi: 10.3390/jcm11061570.

[51] B. C. M. Smits-Engelsman, L. D. Jelsma, and G. D. Ferguson, ‘The Effect of Exergames on Functional Strength, Anaerobic Fitness, Balance and Agility in Children With and Without Motor Coordination Difficulties Living in Low-Income Communities’, Hum. Mov. Sci., vol. 55, pp. 327–337, Oct. 2017, doi: 10.1016/j.humov.2016.07.006.

[52] C. Comeras-Chueca et al., ‘Active Video Games Improve Muscular Fitness and Motor Skills in Children with Overweight or Obesity’, Int. J. Environ. Res. Public. Health, vol. 19, no. 5, Art. no. 5, Jan. 2022, doi: 10.3390/ijerph19052642.

[53] C.-C. Lin, Y.-S. Lin, C.-H. Yeh, C.-C. Huang, L.-C. Kuo, and F.-C. Su, ‘An Exergame-Integrated IoT-Based Ergometer System Delivers Personalized Training Programs for Older Adults and Enhances Physical Fitness: A Pilot Randomized Controlled Trial’, Gerontology, vol. 69, no. 6, pp. 768–782, Jun. 2023, doi: 10.1159/000526951.

[54] É. R. Gouveia et al., ‘The Efficacy of a Multicomponent Functional Fitness Program Based on Exergaming on Cognitive Functioning of Healthy Older Adults: A Randomized Controlled Trial’, J. Aging Phys. Act., vol. 29, no. 4, pp. 586–594, Dec. 2020, doi: 10.1123/japa.2020-0083.

[55] H. Dos Santos, M. D. Bredehoft, F. M. Gonzalez, and S. Montgomery, ‘Exercise Video Games and Exercise Self-Efficacy in Children’, Glob. Pediatr. Health, vol. 3, p. 2333794X16644139, Jan. 2016, doi: 10.1177/2333794X16644139.

[56] S. Bronner, R. Pinsker, R. Naik, and J. A. Noah, ‘Physiological and Psychophysiological Responses to an Exer-Game Training Protocol’, J. Sci. Med. Sport, vol. 19, no. 3, pp. 267–271, Mar. 2016, doi: 10.1016/j.jsams.2015.03.003.

[57] M. D. Kaos et al., ‘Efficacy of Online Multi-Player Versus Single-Player Exergames on Adherence Behaviors Among Children: A Nonrandomized Control Trial’, Ann. Behav. Med., vol. 52, no. 10, pp. 878–889, Sep. 2018, doi: 10.1093/abm/kax061.

[58] G. Hastürk and M. Akyıldız Munusturlar, ‘The Effects of Exergames on Physical and Psychological Health in Young Adults’, Games Health J., vol. 11, no. 6, pp. 425–434, Dec. 2022, doi: 10.1089/g4h.2022.0093.

[61] J. W. Patten, G. Iarocci, and N. Bojin, ‘A Pilot Study of Children’s Physical Activity Levels During Imagination-Based Mobile Games’, J. Child Health Care, vol. 21, no. 3, pp. 292–300, Sep. 2017, doi: 10.1177/1367493517708477.

[64] F. Born, S. Abramowski, and M. Masuch, ‘Exergaming in VR: The Impact of Immersive Embodiment on Motivation, Performance, and Perceived Exertion’, in 2019 11th International Conference on Virtual Worlds and Games for Serious Applications (VS-Games), Sep. 2019, pp. 1–8. doi: 10.1109/VS-Games.2019.8864579.

[67] J. Mueller, D. Niederer, S. Tenberg, L. Oberheim, A. Moesner, and S. Mueller, ‘Acute effects of game-based biofeedback training on trunk motion in chronic low back pain: a randomized cross-over pilot trial’, BMC Sports Sci. Med. Rehabil., vol. 14, no. 1, p. 192, Nov. 2022, doi: 10.1186/s13102-022-00586-z.

[104] K. A. Mackintosh, M. Standage, A. E. Staiano, L. Lester, and M. A. McNarry, ‘Investigating the Physiological and Psychosocial Responses of Single- and Dual-Player Exergaming in Young Adults’, Games Health J., vol. 5, no. 6, pp. 375–381, Dec. 2016, doi: 10.1089/g4h.2016.0015.

[105] D. J. McDonough, Z. C. Pope, N. Zeng, J. E. Lee, and Z. Gao, ‘Comparison of College Students’ Energy Expenditure, Physical Activity, and Enjoyment during Exergaming and Traditional Exercise’, J. Clin. Med., vol. 7, no. 11, Art. no. 11, Nov. 2018, doi: 10.3390/jcm7110433.

[106] D. J. McDonough, Z. C. Pope, N. Zeng, J. E. Lee, and Z. Gao, ‘Retired Elite Athletes’ Physical Activity, Physiological, and Psychosocial Outcomes During Single- and Double-Player Exergaming’, J. Strength Cond. Res., vol. 33, no. 12, pp. 3220–3225, Dec. 2019, doi: 10.1519/JSC.0000000000003386.

[107] Y. Lin, J. Wang, Z. Luo, S. Li, Y. Zhang, and B. C. Wünsche, ‘Dragon Hunter: Loss Aversion for Increasing Physical Activity in AR Exergames’, in Proceedings of the 2023 Australasian Computer Science Week, in ACSW ’23. New York, NY, USA: Association for Computing Machinery, Mar. 2023, pp. 212–221. doi: 10.1145/3579375.3579403.

[119] P.-T. Wu, W.-L. Wu, and I.-H. Chu, ‘Energy Expenditure and Intensity in Healthy Young Adults during Exergaming’, Am. J. Health Behav., vol. 39, no. 4, pp. 556–561, Jul. 2015, doi: 10.5993/AJHB.39.4.12.

[120] K. Hoffmann, D. Sportwiss, S. Hardy, J. Wiemeyer, and S. Göbel, ‘Personalized Adaptive Control of Training Load in Cardio-Exergames—A Feasibility Study’, Games Health J., vol. 4, no. 6, pp. 470–479, Dec. 2015, doi: 10.1089/g4h.2014.0073.

[121] G. A. A. Rodrigues et al., ‘Acute Cardiovascular Responses While Playing Virtual Games Simulated by Nintendo Wii<Sup>®</Sup>’, J. Phys. Ther. Sci., vol. 27, no. 9, pp. 2849–2851, 2015, doi: 10.1589/jpts.27.2849.

[122] V. Benzing, T. Heinks, N. Eggenberger, and M. Schmidt, ‘Acute Cognitively Engaging Exergame-Based Physical Activity Enhances Executive Functions in Adolescents’, PLOS ONE, vol. 11, no. 12, p. e0167501, Dec. 2016, doi: 10.1371/journal.pone.0167501.

[123] T. Moholdt, S. Weie, K. Chorianopoulos, A. I. Wang, and K. Hagen, ‘Exergaming Can Be an Innovative Way of Enjoyable High-Intensity Interval Training’, BMJ Open Sport Exerc. Med., vol. 3, no. 1, p. e000258, Jul. 2017, doi: 10.1136/bmjsem-2017-000258.

[124] A. M. J. Tietjen and G. R. Devereux, ‘Physical Demands of Exergaming in Healthy Young Adults’, J. Strength Cond. Res., vol. 33, no. 7, pp. 1978–1986, Jul. 2019, doi: 10.1519/JSC.0000000000002235.

[125] G. A. A. Rodrigues, P. C. Rodrigues, F. F. da Silva, P. M. Nakamura, W. P. Higino, and R. A. de Souza, ‘Mini-Trampoline Enhances Cardiovascular Responses During a Stationary Running Exergame in Adults’, Biol. Sport, vol. 35, no. 4, pp. 335–342, 2018, doi: 10.5114/biolsport.2018.78052.

[126] R. B. Viana, P. Gentil, M. S. Andrade, R. L. Vancini, and C. A. B. de Lira, ‘Is the Energy Expenditure Provided by Exergames Valid?’, Int. J. Sports Med., vol. 40, no. 9, pp. 563–568, Aug. 2019, doi: 10.1055/a-0955-9394.

[127] C. Roure, D. Pasco, N. Benoît, and L. Deldicque, ‘Impact of a Design-Based Bike Exergame on Young Adults’ Physical Activity Metrics and Situational Interest’, Res. Q. Exerc. Sport, vol. 91, no. 2, pp. 309–315, Apr. 2020, doi: 10.1080/02701367.2019.1665621.

[128] J. Berg and T. Moholdt, ‘Game On: A Cycling Exergame Can Elicit Moderate-To-Vigorous Intensity. A Pilot Study’, BMJ Open Sport Exerc. Med., vol. 6, no. 1, p. e000744, Mar. 2020, doi: 10.1136/bmjsem-2020-000744.

[129] A. L. Martin-Niedecken, T. Schwarz, and A. Schättin, ‘Comparing the Impact of Heart Rate-Based In-Game Adaptations in an Exergame-Based Functional High-Intensity Interval Training on Training Intensity and Experience in Healthy Young Adults’, Front. Psychol., vol. 12, 2021, doi: 10.3389/fpsyg.2021.572877.

[130] C. Aygün and H. Çakir-Atabek, ‘Alternative Model for Physical Activity: Active Video Games Lead to High Physiological Responses’, Res. Q. Exerc. Sport, vol. 0, no. 0, pp. 1–10, Jul. 2021, doi: 10.1080/02701367.2020.1864258.

[131] A. Soria Campo, A. I. Wang, T. Moholdt, and J. Berg, ‘Physiological and Perceptual Responses to Single-player vs. Multiplayer Exergaming’, Front. Sports Act. Living, vol. 4, 2022, doi: 10.3389/fspor.2022.903300.

[132] S. Ketelhut et al., ‘The New Way to Exercise? Evaluating an Innovative Heart-rate-controlled Exergame’, Int. J. Sports Med., vol. 43, no. 1, pp. 77–82, Jan. 2022, doi: 10.1055/a-1520-4742.

[133] D. Marks, L. Rispen, and G. Calara, ‘Greater Physiological Responses While Playing XBox Kinect Compared to Nintendo Wii’, Int. J. Exerc. Sci., vol. 8, no. 2, Apr. 2015, [Online]. Available: https://digitalcommons.wku.edu/ijes/vol8/iss2/7

[134] J. Monedero, E. E. Murphy, and D. J. O’Gorman, ‘Energy expenditure and affect responses to different types of active video game and exercise’, PLOS ONE, vol. 12, no. 5, p. e0176213, May 2017, doi: 10.1371/journal.pone.0176213.

[135] M. Dębska, J. Polechoński, A. Mynarski, and P. Polechoński, ‘Enjoyment and Intensity of Physical Activity in Immersive Virtual Reality Performed on Innovative Training Devices in Compliance with Recommendations for Health’, Int. J. Environ. Res. Public. Health, vol. 16, no. 19, Art. no. 19, Jan. 2019, doi: 10.3390/ijerph16193673.

[136] T. H. Stewart et al., ‘Actual vs. perceived exertion during active virtual reality game exercise’, Front. Rehabil. Sci., vol. 3, p. 887740, 2022, doi: 10.3389/fresc.2022.887740.

[137] M. F. Wouda, J.-A. Gaupseth, E. I. Bengtson, T. Johansen, E. A. Brembo, and E. Lundgaard, ‘Exercise intensity during exergaming in wheelchair-dependent persons with SCI’, Spinal Cord, vol. 61, no. 6, Art. no. 6, Jun. 2023, doi: 10.1038/s41393-023-00893-3.

[138] J. E. Muñoz, A. Goncalves, M. S. Cameirao, S. Bermúdez i Badia, and E. R. Gouveia, ‘Measured and Perceived Physical Responses in Multidimensional Fitness Training through Exergames in Older Adults’, in 2018 10th International Conference on Virtual Worlds and Games for Serious Applications (VS-Games), Sep. 2018, pp. 1–4. doi: 10.1109/VS-Games.2018.8493433.

[139] J. E. Munoz Cardona, M. S. Cameirao, T. Paulino, S. Bermudez i Badia, and E. Rubio, ‘Modulation of Physiological Responses and Activity Levels during Exergame Experiences’, in 2016 8th International Conference on Games and Virtual Worlds for Serious Applications (VS-GAMES), Sep. 2016, pp. 1–8. doi: 10.1109/VS-GAMES.2016.7590353.

[140] H. Liu, Z. Wang, C. Mousas, and D. Kao, ‘Virtual Reality Racket Sports: Virtual Drills for Exercise and Training’, in 2020 IEEE International Symposium on Mixed and Augmented Reality (ISMAR), Nov. 2020, pp. 566–576. doi: 10.1109/ISMAR50242.2020.00084.

[141] E. H. Martin Dantas et al., ‘Neuromotor and Functional Adaptations in Schoolchildren Practicing Exergames’, in 2022 International Conference on Technology Innovations for Healthcare (ICTIH), Sep. 2022, pp. 42–46. doi: 10.1109/ICTIH57289.2022.10111946.

[142] L. Han, Z. Pan, M. Zhang, and F. Tian, ‘A Pleasurable Persuasive Model for E-Fitness System’, in 2016 International Conference on Cyberworlds (CW), Sep. 2016, pp. 89–96. doi: 10.1109/CW.2016.20.

[143] R. Julianjatsono, R. Ferdiana, and R. Hartanto, ‘Development and evaluation of a low cost music based exergame using microsoft kinect’, in 2016 8th International Conference on Information Technology and Electrical Engineering (ICITEE), Oct. 2016, pp. 1–4. doi: 10.1109/ICITEED.2016.7863261.
