# Supplementary material for: Sensing In Exergames for Efficacy and Motion Quality: Scoping Review of Recent Publications
Source: JMIR Serious Games. 2024 Nov 5;12:e52153. doi: 10.2196/52153 (PMC11576609; doi:10.2196/52153)
Supplement: Multimedia Appendix 4 [file games_v12i1e52153_app4.docx]

Multimedia Appendix 4. Summary of Studies with regards to the Evaluation of Games and Motion Sensing.

| Author | Exercises / Games | Exergame Sensors | Exergame Sensors  Categories | | | | Additional Motion Sensing | | |
| --- | --- | --- | --- | --- | --- | --- | --- | --- | --- |
|  |  |  | Bodyworn | Camera | Hybrid | Special | Sensing Technology | Quality Asses-ment | Metric |
| Bronner et al. [56] | Dance Central | XBox 360 Kinect |  | **+** |  |  | Motion Capture (Hybrid) | **+** | PA, mean peak angular displacement of joints (binary encoding) |
| Wu et al. [119] | Kinect Sports (beach volleyball, soccer, boxing, ping-pong, bowling, track & field) | XBox 360 Kinect |  | **+** |  |  |  |  |  |
| McGuire and Willems [36] | Kinect Sports (football, boxing, track & field) | XBox 360 Kinect |  | **+** |  |  |  |  |  |
| Hoffmann et al. [120] | Cycling (playing LetterBird) | Ergometer or Exercise Bike |  |  |  | **+** |  |  |  |
| Rodrigues et al. [121] | Wii Fit Plus | Nintendo Wii Balance Board |  |  |  | **+** |  |  |  |
| Neves et al. [28] | Zumba Fitness Core | XBox 360 Kinect |  | **+** |  |  |  |  |  |
| Dos Santos et al. [55] | Exergame activities from Jackie Chan’s Fitness Studio, Wii Sports, rock-climbing | Jackie Chan’s Fitness Studio: no explicit sensors mentioned or Nintendo Wii |  | **+** | **+** |  |  |  |  |
| Smits-  Engels-  man et al. [51] | Wii Fit (shifting weight, walking on the spot or bending and extending the knees) | Nintendo Wii Balance Board |  |  |  | **+** |  |  |  |
| Barry et al. [46] | Kinect Adventures! (Reflex Ridge, River Rush) and Kinect Sports (Boxing, Super Saver, Target Kick, Bump Bash) against traditional gym-based exercise | XBox 360 Kinect |  | **+** |  |  | force plate | **+** | postural sway |
| Mackintosh et al. [104] | Wii Sports (boxing) | Nintendo Wii |  |  | **+** |  | accelerometer |  | EE |
| Benzing et al. [122] | Shape Up (aerobic exercises such as punches, squats, bench press) & Your Shape: Fitness Evolved (running) | XBox 360 Kinect |  | **+** |  |  |  |  |  |
| Chung et al. [39] | Nintendo Wii Fit U (rowing) against rowing on the indoor rowing machine | Nintendo Wii Balance Board |  |  | **+** |  |  |  |  |
| Moholdt et al. [123] | Cycling (playing Pedal Tanks, multiplayer) | Ergometer or Exercise Bike |  |  |  | **+** | accelerometer |  | PA |
| Patten et al. [61] | Playing on the playground | No sensors |  |  |  |  |  |  |  |
| Tietjen and Devereux [124] | Kinect Adventures! (River Rush) | XBox 360 Kinect |  | **+** |  |  | force plate |  | ground reaction forces |
| Kaos et al. [57] | Cycling (playing Liberi) | Ergometer or Exercise Bike |  |  |  | **+** |  |  |  |
| Pasco et al. [44] | Cycling (playing Greedy Rabbit) | Ergometer or Exercise Bike |  |  |  | **+** | accelerometer |  | PA |
| Farrow et al. [40] | Cycling, leaning head to the left or right to move laterally | Ergometer, HTC Vive |  |  | **+** | **+** |  |  |  |
| McDough et al. [105] | Just Dance & Kinect Adventures! (Reflex Ridge) against treadmill walking | XBox 360 Kinect |  | **+** |  |  | accelerometer |  | PA, EE |
| Rodrigues et al. [125] | Wii Fit Plus (running) | Nintendo Wii Remote (attached at the thigh) to track movements | **+** |  |  |  |  |  |  |
| Polechoński et al. [32] | Your Shape: Fitness Evolved | XBox 360 Kinect |  | **+** |  |  |  |  |  |
| Viana et al. [126] | Hollywood Workout | XBox 360 Kinect |  | **+** |  |  |  |  |  |
| Feodoroff et al. [41] | ICAROS VR flight simulator | ICAROS Home |  |  | **+** | **+** | Surface Electromyography |  | Muscle Activity |
| McDough et al. [106] | Kinect Adventures! (Reflex Ridge, single and multiplayer) | XBox 360 Kinect |  | **+** |  |  | accelerometer |  | PA, EE, steps |
| Çakir-Atabek et al. [33] | Dance Central 3 (Fitness mode), Fighters Uncaged | XBox 360 Kinect |  | **+** |  |  |  |  |  |
| Roure et al. [127] | Cycling (playing Greedy Rabbit) | Ergometer or Exercise Bike |  |  |  | **+** | Exercise Bike |  | cadence (in rpm) |
| Berg and Moholdt [128] | Cycling (playing Pedal Tanks, multiplayer) | Ergometer or Exercise Bike |  |  |  | **+** |  |  |  |
| Berg et al. [49] | Cycling (playing Pedal Tanks, multiplayer) | Ergometer or Exercise Bike |  |  |  | **+** | accelerometer |  | MET (EE) |
| Ko et al. [42] | Skiing | SKI Fit 360 |  |  |  | **+** | IMU (body-worn) | **+** | range of motion |
| Martin-Niedecken et al. [129] | Touch, squat, jump, punch, lunge, skipping, burpee | The ExerCube |  |  | **+** |  |  |  |  |
| Aygün and Çakir-Atabek [130] | Dancing and fighting exergames against resting, watching TV, and brisk walking | XBox 360 Kinect |  | **+** |  |  |  |  |  |
| Ketelhut et al. [38] | Touch, squat, jump, punch, lunge, skipping, burpee | The ExerCube |  |  | **+** |  |  |  | Blood lactate enzymatic amperometry |
| Comeras-Chueca et al. [52] | Kinect Adventures!, Kinect Sport, Wii Sports, Just Dance, Mario and Sonic at the Olympic Games, Dance Dance Revolution, BKOOL cycling simulator | XBox 360 Kinect, Nintendo Wii, Nintendo Wii dance mats, ergometer |  | **+** |  | **+** | accelerometer, force plate,  handgrip dynamometer | **+** | PA, Muscular Fitness,  Max Isometric  Strength, Handgrip  Strength (between exercises),  Counter Movement Jump |
| Berg et al. [29] | Cycling | Ergometer or Exercise Bike |  |  |  | **+** |  |  |  |
| Badau et al. [23] | Custom exercise for reaction time | Fitlight System |  |  |  | **+** |  |  |  |
| Soria Campo et al. [131] | Cycling | Ergometer or Exercise Bike |  |  |  | **+** |  |  |  |
| Gouveia et al. [54] | Grape stomping, rabelos, exermusic, toboggan, ride, exerpong | XBox 360 Kinect |  | **+** |  |  |  |  |  |
| Ketelhut et al. [132] | Touch, squat, jump, punch, lunge, skipping, burpee | The ExerCube |  |  | **+** |  |  |  |  |
| Li et al. [27] | Bowling | XBox 360 Kinect, Wii |  | **+** | **+** |  | Camera + Screen Protractor | **+** | Shoulder Flexibility |
| Kircher et al. [35] | Touch, squat, jump, punch, lunge, skipping, burpee | The ExerCube |  |  | **+** |  |  |  |  |
| Martin-Niedecken et al. [92] | Touch, squat, jump, punch, lunge, skipping, burpee | The ExerCube |  |  | **+** |  |  |  |  |
| Marks et al. [133] | Kinect / Wii Just Dance 2, Kinect Boxing, Wii Boxin | XBox 360 Kinect, Wii |  | **+** | **+** |  |  |  |  |
| Evans et al. [34] | Beat Saber, Holopoint, Hot Squat, Relax Walk | HTC Vive |  |  | **+** |  | Accelerometer |  |  |
| Sousa et al. [43] | Beat Saber, Thumper | HTC Vive |  |  | **+** |  | Accelerometer |  | frequency, duration, PA |
| Wu et al. [47] | Ring Fit Adventure | Nintendo Switch | **+** |  |  |  | IMU (body-worn) |  | peak acceleration/s, PA |
| Monedero et al. [134] | Kinect Adventures, Your Shape FItness Evolve | XBox 360 Kinect |  | **+** |  |  |  |  |  |
| Dębska et al. [135] | Travr Training OPS, Flight | Omni Treadmill, Icaros Pro flight simulator |  |  | **+** | **+** |  |  |  |
| Ketelhut et al. [50] | Touch, squat, jump, punch, lunge, skipping, burpee | The ExerCube |  |  | **+** |  | Camera system |  | Physical Fitness  (between exercises),  Counter Movement Jump |
| Lisón et al. [31] | Kinect Adventures | XBox 360 Kinect |  | **+** |  |  |  |  |  |
| Stewart et al. [136] | Fruit Ninja VR, Beat Saber, Holopoint | HTC Vive |  |  | **+** |  |  |  |  |
| Mueller et al. [67] | Valedo Home | Valedo Home | **+** |  |  |  | Motion Capture (Hybrid) | **+** | trunk lateral angle |
| Ciążyńska et al. [37] | Audio Trip | Oculus Rift | **+** |  | **+** |  |  |  |  |
| Lin et al. [53] | EIoT-Ergo | Ergometer |  |  |  | **+** |  |  |  |
| Lin et al. [107] | Dragon Hunter | Smartphone GPS + pedometer | **+** |  |  |  |  |  |  |
| Wounda et al. [137] | Fruit Ninja VR, Wii Sports Boxing, VR Boxing | XBox 360 Kinect, Nintendo Wii, Oculus Rift |  | **+** | **+** |  |  |  |  |
| Mologne et al. [30] | Tower Defense | Black Box VR + HTC Vive |  |  | **+** | **+** |  |  |  |
| Hastürk and Munusturlar [58] | Kinect Adventure: Reflex Ridge, Dance Central 3, Kinect Sports 3: Volleyball | XBox 360 Kinect |  | **+** |  |  | force plate, LED-based  reaction time measurement |  |  |
| Sheu et al. [48] | EverGreen Fitness System (3 different games) | XBox 360 Kinect |  | **+** |  |  |  |  |  |
| Rosly et al. [45] | Move Boxing, Move Kayaking; optionally with additional weights | PS3 Move |  | **+** |  |  | Camera | **+** | subjective analysis of gross upper body biomechanics (by an expert) |
| Wünsche et al. [22] | Rift Racers (Cycling exergame) | Ergometer |  |  |  | **+** |  |  |  |
| Muñoz et al. [138] | Custom Kinect Games | XBox 360 Kinect |  | **+** |  |  |  |  |  |
| Cardona et al. [139] | Exerpong | XBox 360 Kinect |  | **+** |  |  |  |  |  |
| Liu et al. [140] | Custom VR table tennis game | HTC Vive Pro |  |  | **+** |  |  |  |  |
| Liu et al. [140] | Custom VR table tennis game | HTC Vive Pro |  |  | **+** |  |  |  |  |
| Martin Dantas et al. [141] | Dance Dance Revolution | XBox 360 Kinect |  | **+** |  |  |  |  |  |
| Han et al. [142] | Custom ergometer game | Ergometer |  |  |  | **+** |  |  |  |
| Julainjatsono et al. [143] | Beat-Beat Fitness | XBox 360 Kinect |  | **+** |  |  |  |  |  |
| Total |  |  | 5 | 28 | 22 | 21 |  | 7 |  |

Abbreviations: HR, Heart Rate; VO2, Oxygen Uptake; EE, Energy Expenditure; MET, Metabolic Equivalent of Task, PA, Physical Activity

**References**

[22] B. C. Wünsche et al., ‘Rift Racers - Effect of Balancing and Competition on Exertion, Enjoyment, and Motivation in an Immersive Exergame’, in 2021 36th International Conference on Image and Vision Computing New Zealand (IVCNZ), Dec. 2021, pp. 1–6. doi: 10.1109/IVCNZ54163.2021.9653159.

[23] D. Badau et al., ‘The Impact of Implementing an Exergame Program on the Level of Reaction Time Optimization in Handball, Volleyball, and Basketball Players’, Int. J. Environ. Res. Public. Health, vol. 19, no. 9, Art. no. 9, Jan. 2022, doi: 10.3390/ijerph19095598.

[27] J. Li, L. Li, P. Huo, C. Ma, L. Wang, and Y. L. Theng, ‘Wii or Kinect? A Pilot Study of the Exergame Effects on Older Adults’ Physical Fitness and Psychological Perception’, Int. J. Environ. Res. Public. Health, vol. 18, no. 24, Art. no. 24, Jan. 2021, doi: 10.3390/ijerph182412939.

[28] L. E. D. S. Neves et al., ‘Cardiovascular Effects of Zumba® Performed in a Virtual Environment Using Xbox Kinect’, J. Phys. Ther. Sci., vol. 27, no. 9, pp. 2863–2865, 2015, doi: 10.1589/jpts.27.2863.

[29] J. Berg, G. Haugen, A. I. Wang, and T. Moholdt, ‘High-Intensity Exergaming for Improved Cardiorespiratory Fitness: A Randomised, Controlled Trial’, Eur. J. Sport Sci., vol. 22, no. 6, pp. 867–876, Jun. 2022, doi: 10.1080/17461391.2021.1921852.

[30] M. S. Mologne et al., ‘The Efficacy of an Immersive Virtual Reality Exergame Incorporating an Adaptive Cable Resistance System on Fitness and Cardiometabolic Measures: A 12-Week Randomized Controlled Trial’, Int. J. Environ. Res. Public. Health, vol. 20, no. 1, Art. no. 1, Jan. 2023, doi: 10.3390/ijerph20010210.

[31] J. F. Lisón et al., ‘Competitive active video games: Physiological and psychological responses in children and adolescents’, Paediatr. Child Health, vol. 20, no. 7, pp. 373–376, Oct. 2015, doi: 10.1093/pch/20.7.373.

[32] J. Polechoński, M. Dębska, and P. G. Dębski, ‘Exergaming Can Be a Health-Related Aerobic Physical Activity’, BioMed Res. Int., vol. 2019, p. e1890527, Jun. 2019, doi: 10.1155/2019/1890527.

[33] H. Çakir-Atabek, C. Aygün, and B. Dokumacı, ‘Active Video Games Versus Traditional Exercises: Energy Expenditure and Blood Lactate Responses’, Res. Q. Exerc. Sport, vol. 91, no. 2, pp. 188–196, Apr. 2020, doi: 10.1080/02701367.2019.1653431.

[34] E. Evans, K. E. Naugle, A. S. Kaleth, B. Arnold, and K. M. Naugle, ‘Physical Activity Intensity, Perceived Exertion, and Enjoyment During Head-Mounted Display Virtual Reality Games’, Games Health J., vol. 10, no. 5, pp. 314–320, Oct. 2021, doi: 10.1089/g4h.2021.0036.

[35] E. Kircher et al., ‘Acute Effects of Heart Rate-Controlled Exergaming on Vascular Function in Young Adults’, Games Health J., vol. 11, no. 1, pp. 58–66, Feb. 2022, doi: 10.1089/g4h.2021.0196.

[36] S. McGuire and M. E. Willems, ‘Physiological Responses During Multiplay Exergaming in Young Adult Males are Game-Dependent’, J. Hum. Kinet., vol. 46, pp. 263–271, Jul. 2015, doi: 10.1515/hukin-2015-0054.

[37] J. Ciążyńska and J. Maciaszek, ‘Effects of Low-Immersive vs. High-Immersive Exercise Environment on Postural Stability and Reaction and Motor Time of Healthy Young Adults’, J. Clin. Med., vol. 12, no. 1, Art. no. 1, Jan. 2023, doi: 10.3390/jcm12010389.

[38] S. Ketelhut et al., ‘Gaming Instead of Training? Exergaming Induces High-Intensity Exercise Stimulus and Reduces Cardiovascular Reactivity to Cold Pressor Test’, Front. Cardiovasc. Med., vol. 9, 2022, doi: 10.3389/fcvm.2022.798149.

[39] L. M. Y. Chung, F. H. Sun, and C. T. M. Cheng, ‘Physiological and Perceived Responses in Different Levels of Exergames in Elite Athletes’, Games Health J., vol. 6, no. 1, pp. 57–60, Feb. 2017, doi: 10.1089/g4h.2016.0074.

[40] M. Farrow, C. Lutteroth, P. C. Rouse, and J. L. J. Bilzon, ‘Virtual-Reality Exergaming Improves Performance During High-Intensity Interval Training’, Eur. J. Sport Sci., vol. 19, no. 6, pp. 719–727, Jul. 2019, doi: 10.1080/17461391.2018.1542459.

[41] B. Feodoroff, I. Konstantinidis, and I. Froböse, ‘Effects of Full Body Exergaming in Virtual Reality on Cardiovascular and Muscular Parameters: Cross-Sectional Experiment’, JMIR Serious Games, vol. 7, no. 3, p. e12324, Aug. 2019, doi: 10.2196/12324.

[42] J. Ko, S.-W. Jang, H. T. Lee, H.-K. Yun, and Y. S. Kim, ‘Effects of Virtual Reality and Non–Virtual Reality Exercises on the Exercise Capacity and Concentration of Users in a Ski Exergame: Comparative Study’, JMIR Serious Games, vol. 8, no. 4, p. e16693, Oct. 2020, doi: 10.2196/16693.

[43] C. V. Sousa et al., ‘Active video games in fully immersive virtual reality elicit moderate-to-vigorous physical activity and improve cognitive performance in sedentary college students’, J. Sport Health Sci., vol. 11, no. 2, pp. 164–171, Mar. 2022, doi: 10.1016/j.jshs.2021.05.002.

[44] D. Pasco, C. Roure, G. Kermarrec, Z. Pope, and Z. Gao, ‘The Effects of a Bike Active Video Game on Players’ Physical Activity and Motivation’, J. Sport Health Sci., vol. 6, no. 1, pp. 25–32, Mar. 2017, doi: 10.1016/j.jshs.2016.11.007.

[45] M. M. Rosly, M. Halaki, H. M. Rosly, N. Hasnan, R. Husain, and G. M. Davis, ‘Arm Exercises for Individuals with Spinal Cord Injury: Exergaming versus Arm Cranking’, in 2019 IEEE 7th International Conference on Serious Games and Applications for Health (SeGAH), Aug. 2019, pp. 1–7. doi: 10.1109/SeGAH.2019.8882460.

[46] G. Barry, P. van Schaik, A. MacSween, J. Dixon, and D. Martin, ‘Exergaming (Xbox KinectTM) Versus Traditional Gym-Based Exercise for Postural Control, Flow and Technology Acceptance in Healthy Adults: A Randomised Controlled Trial’, BMC Sports Sci. Med. Rehabil., vol. 8, no. 1, p. 25, Aug. 2016, doi: 10.1186/s13102-016-0050-0.

[47] Y.-S. Wu et al., ‘Effect of the Nintendo Ring Fit Adventure Exergame on Running Completion Time and Psychological Factors Among University Students Engaging in Distance Learning During the COVID-19 Pandemic: Randomized Controlled Trial’, JMIR Serious Games, vol. 10, no. 1, p. e35040, Mar. 2022, doi: 10.2196/35040.

[48] F.-R. Sheu, Y.-L. Lee, H.-T. Hsu, and N.-S. Chen, ‘Effects of Gesture-Based Fitness Games on Functional Fitness of the Elders’, in 2015 IEEE 15th International Conference on Advanced Learning Technologies, Jul. 2015, pp. 158–160. doi: 10.1109/ICALT.2015.35.

[49] J. Berg, A. I. Wang, S. Lydersen, and T. Moholdt, ‘Can Gaming Get You Fit?’, Front. Physiol., vol. 11, 2020, doi: 10.3389/fphys.2020.01017.

[50] S. Ketelhut, L. Röglin, A. L. Martin-Niedecken, C. R. Nigg, and K. Ketelhut, ‘Integrating Regular Exergaming Sessions in the ExerCube into a School Setting Increases Physical Fitness in Elementary School Children: A Randomized Controlled Trial’, J. Clin. Med., vol. 11, no. 6, Art. no. 6, Jan. 2022, doi: 10.3390/jcm11061570.

[51] B. C. M. Smits-Engelsman, L. D. Jelsma, and G. D. Ferguson, ‘The Effect of Exergames on Functional Strength, Anaerobic Fitness, Balance and Agility in Children With and Without Motor Coordination Difficulties Living in Low-Income Communities’, Hum. Mov. Sci., vol. 55, pp. 327–337, Oct. 2017, doi: 10.1016/j.humov.2016.07.006.

[52] C. Comeras-Chueca et al., ‘Active Video Games Improve Muscular Fitness and Motor Skills in Children with Overweight or Obesity’, Int. J. Environ. Res. Public. Health, vol. 19, no. 5, Art. no. 5, Jan. 2022, doi: 10.3390/ijerph19052642.

[53] C.-C. Lin, Y.-S. Lin, C.-H. Yeh, C.-C. Huang, L.-C. Kuo, and F.-C. Su, ‘An Exergame-Integrated IoT-Based Ergometer System Delivers Personalized Training Programs for Older Adults and Enhances Physical Fitness: A Pilot Randomized Controlled Trial’, Gerontology, vol. 69, no. 6, pp. 768–782, Jun. 2023, doi: 10.1159/000526951.

[54] É. R. Gouveia et al., ‘The Efficacy of a Multicomponent Functional Fitness Program Based on Exergaming on Cognitive Functioning of Healthy Older Adults: A Randomized Controlled Trial’, J. Aging Phys. Act., vol. 29, no. 4, pp. 586–594, Dec. 2020, doi: 10.1123/japa.2020-0083.

[55] H. Dos Santos, M. D. Bredehoft, F. M. Gonzalez, and S. Montgomery, ‘Exercise Video Games and Exercise Self-Efficacy in Children’, Glob. Pediatr. Health, vol. 3, p. 2333794X16644139, Jan. 2016, doi: 10.1177/2333794X16644139.

[56] S. Bronner, R. Pinsker, R. Naik, and J. A. Noah, ‘Physiological and Psychophysiological Responses to an Exer-Game Training Protocol’, J. Sci. Med. Sport, vol. 19, no. 3, pp. 267–271, Mar. 2016, doi: 10.1016/j.jsams.2015.03.003.

[57] M. D. Kaos et al., ‘Efficacy of Online Multi-Player Versus Single-Player Exergames on Adherence Behaviors Among Children: A Nonrandomized Control Trial’, Ann. Behav. Med., vol. 52, no. 10, pp. 878–889, Sep. 2018, doi: 10.1093/abm/kax061.

[58] G. Hastürk and M. Akyıldız Munusturlar, ‘The Effects of Exergames on Physical and Psychological Health in Young Adults’, Games Health J., vol. 11, no. 6, pp. 425–434, Dec. 2022, doi: 10.1089/g4h.2022.0093.

[61] J. W. Patten, G. Iarocci, and N. Bojin, ‘A Pilot Study of Children’s Physical Activity Levels During Imagination-Based Mobile Games’, J. Child Health Care, vol. 21, no. 3, pp. 292–300, Sep. 2017, doi: 10.1177/1367493517708477.

[67] J. Mueller, D. Niederer, S. Tenberg, L. Oberheim, A. Moesner, and S. Mueller, ‘Acute effects of game-based biofeedback training on trunk motion in chronic low back pain: a randomized cross-over pilot trial’, BMC Sports Sci. Med. Rehabil., vol. 14, no. 1, p. 192, Nov. 2022, doi: 10.1186/s13102-022-00586-z.

[92] A. L. Martin-Niedecken, K. Rogers, L. Turmo Vidal, E. D. Mekler, and E. Márquez Segura, ‘ExerCube vs. Personal Trainer: Evaluating a Holistic, Immersive, and Adaptive Fitness Game Setup’, in Proceedings of the 2019 CHI Conference on Human Factors in Computing Systems, in CHI ’19. New York, NY, USA: Association for Computing Machinery, May 2019, pp. 1–15. doi: 10.1145/3290605.3300318.

[104] K. A. Mackintosh, M. Standage, A. E. Staiano, L. Lester, and M. A. McNarry, ‘Investigating the Physiological and Psychosocial Responses of Single- and Dual-Player Exergaming in Young Adults’, Games Health J., vol. 5, no. 6, pp. 375–381, Dec. 2016, doi: 10.1089/g4h.2016.0015.

[105] D. J. McDonough, Z. C. Pope, N. Zeng, J. E. Lee, and Z. Gao, ‘Comparison of College Students’ Energy Expenditure, Physical Activity, and Enjoyment during Exergaming and Traditional Exercise’, J. Clin. Med., vol. 7, no. 11, Art. no. 11, Nov. 2018, doi: 10.3390/jcm7110433.

[106] D. J. McDonough, Z. C. Pope, N. Zeng, J. E. Lee, and Z. Gao, ‘Retired Elite Athletes’ Physical Activity, Physiological, and Psychosocial Outcomes During Single- and Double-Player Exergaming’, J. Strength Cond. Res., vol. 33, no. 12, pp. 3220–3225, Dec. 2019, doi: 10.1519/JSC.0000000000003386.

[107] Y. Lin, J. Wang, Z. Luo, S. Li, Y. Zhang, and B. C. Wünsche, ‘Dragon Hunter: Loss Aversion for Increasing Physical Activity in AR Exergames’, in Proceedings of the 2023 Australasian Computer Science Week, in ACSW ’23. New York, NY, USA: Association for Computing Machinery, Mar. 2023, pp. 212–221. doi: 10.1145/3579375.3579403.

[119] P.-T. Wu, W.-L. Wu, and I.-H. Chu, ‘Energy Expenditure and Intensity in Healthy Young Adults during Exergaming’, Am. J. Health Behav., vol. 39, no. 4, pp. 556–561, Jul. 2015, doi: 10.5993/AJHB.39.4.12.

[120] K. Hoffmann, D. Sportwiss, S. Hardy, J. Wiemeyer, and S. Göbel, ‘Personalized Adaptive Control of Training Load in Cardio-Exergames—A Feasibility Study’, Games Health J., vol. 4, no. 6, pp. 470–479, Dec. 2015, doi: 10.1089/g4h.2014.0073.

[121] G. A. A. Rodrigues et al., ‘Acute Cardiovascular Responses While Playing Virtual Games Simulated by Nintendo Wii<Sup>®</Sup>’, J. Phys. Ther. Sci., vol. 27, no. 9, pp. 2849–2851, 2015, doi: 10.1589/jpts.27.2849.

[122] V. Benzing, T. Heinks, N. Eggenberger, and M. Schmidt, ‘Acute Cognitively Engaging Exergame-Based Physical Activity Enhances Executive Functions in Adolescents’, PLOS ONE, vol. 11, no. 12, p. e0167501, Dec. 2016, doi: 10.1371/journal.pone.0167501.

[123] T. Moholdt, S. Weie, K. Chorianopoulos, A. I. Wang, and K. Hagen, ‘Exergaming Can Be an Innovative Way of Enjoyable High-Intensity Interval Training’, BMJ Open Sport Exerc. Med., vol. 3, no. 1, p. e000258, Jul. 2017, doi: 10.1136/bmjsem-2017-000258.

[124] A. M. J. Tietjen and G. R. Devereux, ‘Physical Demands of Exergaming in Healthy Young Adults’, J. Strength Cond. Res., vol. 33, no. 7, pp. 1978–1986, Jul. 2019, doi: 10.1519/JSC.0000000000002235.

[125] G. A. A. Rodrigues, P. C. Rodrigues, F. F. da Silva, P. M. Nakamura, W. P. Higino, and R. A. de Souza, ‘Mini-Trampoline Enhances Cardiovascular Responses During a Stationary Running Exergame in Adults’, Biol. Sport, vol. 35, no. 4, pp. 335–342, 2018, doi: 10.5114/biolsport.2018.78052.

[126] R. B. Viana, P. Gentil, M. S. Andrade, R. L. Vancini, and C. A. B. de Lira, ‘Is the Energy Expenditure Provided by Exergames Valid?’, Int. J. Sports Med., vol. 40, no. 9, pp. 563–568, Aug. 2019, doi: 10.1055/a-0955-9394.

[127] C. Roure, D. Pasco, N. Benoît, and L. Deldicque, ‘Impact of a Design-Based Bike Exergame on Young Adults’ Physical Activity Metrics and Situational Interest’, Res. Q. Exerc. Sport, vol. 91, no. 2, pp. 309–315, Apr. 2020, doi: 10.1080/02701367.2019.1665621.

[128] J. Berg and T. Moholdt, ‘Game On: A Cycling Exergame Can Elicit Moderate-To-Vigorous Intensity. A Pilot Study’, BMJ Open Sport Exerc. Med., vol. 6, no. 1, p. e000744, Mar. 2020, doi: 10.1136/bmjsem-2020-000744.

[129] A. L. Martin-Niedecken, T. Schwarz, and A. Schättin, ‘Comparing the Impact of Heart Rate-Based In-Game Adaptations in an Exergame-Based Functional High-Intensity Interval Training on Training Intensity and Experience in Healthy Young Adults’, Front. Psychol., vol. 12, 2021, doi: 10.3389/fpsyg.2021.572877.

[130] C. Aygün and H. Çakir-Atabek, ‘Alternative Model for Physical Activity: Active Video Games Lead to High Physiological Responses’, Res. Q. Exerc. Sport, vol. 0, no. 0, pp. 1–10, Jul. 2021, doi: 10.1080/02701367.2020.1864258.

[131] A. Soria Campo, A. I. Wang, T. Moholdt, and J. Berg, ‘Physiological and Perceptual Responses to Single-player vs. Multiplayer Exergaming’, Front. Sports Act. Living, vol. 4, 2022, doi: 10.3389/fspor.2022.903300.

[132] S. Ketelhut et al., ‘The New Way to Exercise? Evaluating an Innovative Heart-rate-controlled Exergame’, Int. J. Sports Med., vol. 43, no. 1, pp. 77–82, Jan. 2022, doi: 10.1055/a-1520-4742.

[133] D. Marks, L. Rispen, and G. Calara, ‘Greater Physiological Responses While Playing XBox Kinect Compared to Nintendo Wii’, Int. J. Exerc. Sci., vol. 8, no. 2, Apr. 2015, [Online]. Available: https://digitalcommons.wku.edu/ijes/vol8/iss2/7

[134] J. Monedero, E. E. Murphy, and D. J. O’Gorman, ‘Energy expenditure and affect responses to different types of active video game and exercise’, PLOS ONE, vol. 12, no. 5, p. e0176213, May 2017, doi: 10.1371/journal.pone.0176213.

[135] M. Dębska, J. Polechoński, A. Mynarski, and P. Polechoński, ‘Enjoyment and Intensity of Physical Activity in Immersive Virtual Reality Performed on Innovative Training Devices in Compliance with Recommendations for Health’, Int. J. Environ. Res. Public. Health, vol. 16, no. 19, Art. no. 19, Jan. 2019, doi: 10.3390/ijerph16193673.

[136] T. H. Stewart et al., ‘Actual vs. perceived exertion during active virtual reality game exercise’, Front. Rehabil. Sci., vol. 3, p. 887740, 2022, doi: 10.3389/fresc.2022.887740.

[137] M. F. Wouda, J.-A. Gaupseth, E. I. Bengtson, T. Johansen, E. A. Brembo, and E. Lundgaard, ‘Exercise intensity during exergaming in wheelchair-dependent persons with SCI’, Spinal Cord, vol. 61, no. 6, Art. no. 6, Jun. 2023, doi: 10.1038/s41393-023-00893-3.

[138] J. E. Muñoz, A. Goncalves, M. S. Cameirao, S. Bermúdez i Badia, and E. R. Gouveia, ‘Measured and Perceived Physical Responses in Multidimensional Fitness Training through Exergames in Older Adults’, in 2018 10th International Conference on Virtual Worlds and Games for Serious Applications (VS-Games), Sep. 2018, pp. 1–4. doi: 10.1109/VS-Games.2018.8493433.

[139] J. E. Munoz Cardona, M. S. Cameirao, T. Paulino, S. Bermudez i Badia, and E. Rubio, ‘Modulation of Physiological Responses and Activity Levels during Exergame Experiences’, in 2016 8th International Conference on Games and Virtual Worlds for Serious Applications (VS-GAMES), Sep. 2016, pp. 1–8. doi: 10.1109/VS-GAMES.2016.7590353.

[140] H. Liu, Z. Wang, C. Mousas, and D. Kao, ‘Virtual Reality Racket Sports: Virtual Drills for Exercise and Training’, in 2020 IEEE International Symposium on Mixed and Augmented Reality (ISMAR), Nov. 2020, pp. 566–576. doi: 10.1109/ISMAR50242.2020.00084.

[141] E. H. Martin Dantas et al., ‘Neuromotor and Functional Adaptations in Schoolchildren Practicing Exergames’, in 2022 International Conference on Technology Innovations for Healthcare (ICTIH), Sep. 2022, pp. 42–46. doi: 10.1109/ICTIH57289.2022.10111946.

[142] L. Han, Z. Pan, M. Zhang, and F. Tian, ‘A Pleasurable Persuasive Model for E-Fitness System’, in 2016 International Conference on Cyberworlds (CW), Sep. 2016, pp. 89–96. doi: 10.1109/CW.2016.20.

[143] R. Julianjatsono, R. Ferdiana, and R. Hartanto, ‘Development and evaluation of a low cost music based exergame using microsoft kinect’, in 2016 8th International Conference on Information Technology and Electrical Engineering (ICITEE), Oct. 2016, pp. 1–4. doi: 10.1109/ICITEED.2016.7863261.
